# Halogenation dictates the architecture of amyloid peptide nanostructures†
†Electronic supplementary information (ESI) available. See DOI: 10.1039/c7nr03263c


**DOI:** 10.1039/c7nr03263c

**Published:** 2017-06-22

**Authors:** Andrea Pizzi, Claudia Pigliacelli, Alessandro Gori, Nonappa, Olli Ikkala, Nicola Demitri, Giancarlo Terraneo, Valeria Castelletto, Ian W. Hamley, Francesca Baldelli Bombelli, Pierangelo Metrangolo

**Affiliations:** a Laboratory of Supramolecular and BioNano Materials (SupraBioNanoLab) , Department of Chemistry , Materials , and Chemical Engineering “Giulio Natta” , Politecnico di Milano , Via Luigi Mancinelli 7 , Milano I-20131 , Italy . Email: pierangelo.metrangolo@polimi.it; b Department of Applied Physics , Aalto University , Espoo , FI-02150 , Finland; c Istituto di Chimica del Riconoscimento Molecolare – National Research Council of Italy (ICRM-CNR) , Laboratory of Peptide and Protein Chemistry , Via Mario Bianco 9 , 20131 Milano , Italy; d Elettra – Sincrotrone Trieste , S.S. 14 Km 163.5 in Area Science Park , 34149 Basovizza – Trieste , Italy; e Department of Chemistry , University of Reading , Whiteknights , Reading , RG6 6AD , UK

## Abstract

Amyloid peptides yield a plethora of interesting nanostructures though difficult to control. Here we report that depending on the number, position, and nature of the halogen atoms introduced into either one or both phenylalanine benzene rings of the amyloid β peptide-derived core-sequence KLVFF, four different architectures were obtained in a controlled manner. Our findings demonstrate that halogenation may develop as a general strategy to engineer amyloidal peptide self-assembly and obtain new amyloidal nanostructures.

## Abstract 2

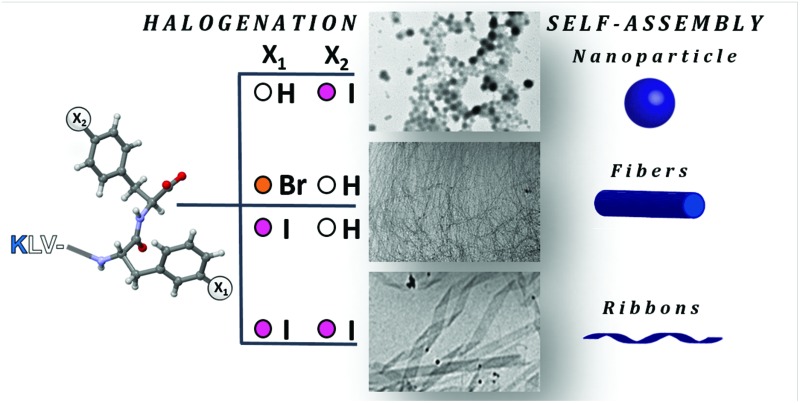
Upon changing the position, nature and number of the halogen atoms, the same amyloidogenic peptide self-assembles into different nanostructures.

Besides pathological roles in many diseases, *e.g.*, Alzheimer's, Parkinson's, Creutzfeldt–Jakob, and Huntington's,^1^ amyloid peptide architectures have found many other nonbiological applications^2^,^3^ such as forming highly ordered nanomaterials.^4^ Together with their biocompatibility and the ease of production,^5^ amyloidogenic peptides show a very versatile polymorphic behavior yielding a broad range of hierarchical structures, such as tapes, ribbons, fibers, nanoparticles, and nanotubes.^6^–^9^ Subtle variations in the experimental conditions, peptide sequence or its chemical functionalization may impact the self-assembly pathway and, consequently, the resulting nanostructures.^10^

Despite representing a powerful tool to produce various nanoobjects, amyloid intrinsic polymorphism may, however, limit the controlled construction of specifically designed nanostructures. The possibility of tuning such polymorphic behavior is still in its early stages.^11^,^12^ For example, an amino acid sequence in constitutionally isomeric tetrapeptide amphiphiles has recently been shown to dictate nanostructural architecture.^13^ The chemical functionalization of the peptide sequence has also been demonstrated to be a powerful tool for controlling amyloid self-assembly.^14^,^15^ In this regard, polymer conjugation has been studied extensively as a particularly fruitful strategy.^16^

Recently, some of us demonstrated that halogenation at the *p*-position of either one or both phenylalanine (Phe) benzene rings of the human calcitonin-derived fibrillogenic peptide sequence DFNKF^17^ promotes amyloid self-assembly. The hydrogel formation efficiency is also promoted by a rich variety of noncovalent interactions given by halogen atoms,^18^ among these, the halogen bond.^19^ In this context, here we applied this strategy to dictate the architecture of the obtained amyloid nanostructures starting from the amyloid β (Aβ) peptide-derived core-sequence KLVFF (H_2_N-Lys-Leu-Val-Phe-Phe-COOH) (Fig. 1). Thanks to the presence of the –FF– motif, this pentapeptide has a highly pronounced aggregation propensity, as proved both computationally^20^–^22^ and experimentally.^23^

**Fig. 1:**
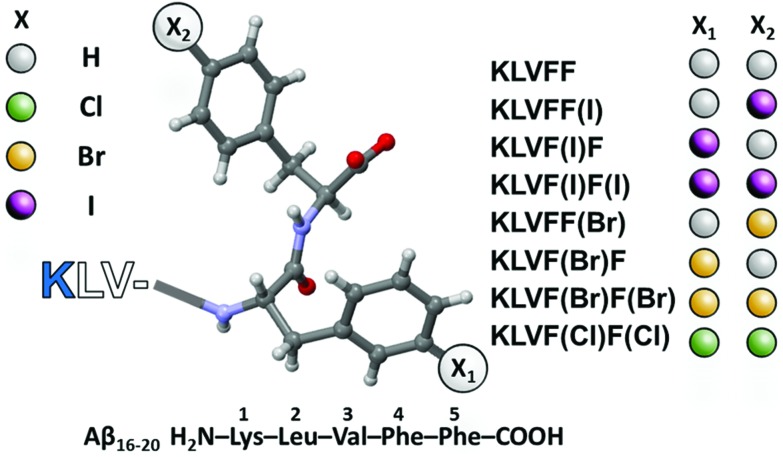
Chemical structures of halogenated KLVFF peptides.

By single-atom hydrogen-for-halogen replacement at the *p*-position of either one or both Phe benzene rings of KLVFF, we demonstrate that halogenation dictates the controlled formation of various nanostructures. Nanoparticles, ribbons, fibrils, and “cotton balls” are specifically obtained depending on the number, position, and nature of the introduced halogen atoms. This is remarkable for such a modest chemical structure modification. These results reveal the potential of controlling the morphology of amyloid nanostructures through the single-point halogenation of the amino acid sequence.

To study the effect of the introduction of halogen atoms at specific positions of the KLVFF pentapeptide on their self-assembly, we obtained the 4-*p*-X-Phe derivative (halogenation on inner phenylalanine), the 5-*p*-X-Phe derivative (halogenation on terminal phenylalanine), as well as the 4,5-bis-*p*-X-Phe derivative (halogenation on both phenylalanine residues), where X = I, Br, Cl, H (Fig. 1). Importantly, all of the studied peptides carry free amino (N) and carboxyl (C) termini.

The aqueous samples of the peptides were prepared by direct dispersion in Milli-Q water (see the ESI†). As an indication of the formation of fibrils,^22^ hydrogelation tests were performed and minimum gelation concentrations (MGCs) were determined. KLVF(I)F, KLVF(Br)F, and KLVF(I)F(I) were found to form gels (Fig. 2) above MGCs that were much lower than the wild-type fragment KLVFF (60 mM) (Table S1†).^23^ The best gelator of the series, KLVF(I)F, showed an MGC of 7 mM (0.5% w/w, *i.e.*, 8-fold lower than KLVFF). This indicates that halogenation has a clear effect on the peptide self-assembly process, promoting fibrillation, as previously observed.^17^

**Fig. 2:**
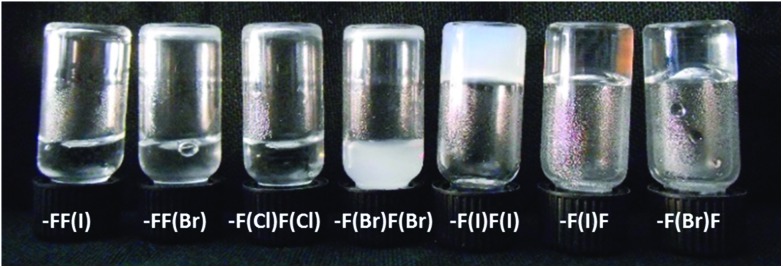
15 mM peptide water samples upon aging 48 h at r.t.

A working concentration of 15 mM was chosen to compare all the gel-forming halogenated peptides under the same conditions (Table S2†). The efficiency of gel formation under these conditions followed the order KLVF(I)F > KLVF(Br)F > KLVF(I)F(I). The first two mono-halogenated peptides yielded homogeneous and transparent gels. The bis-iodinated one, instead, formed a homogeneously opaque, off-whitish, cloudy gel.

The characterization of the different halogenated peptide hydrogels was done by oscillatory rheology (ring-cast method) using the 15 mM concentration samples. The mono-iodinated peptide was confirmed to form the stiffest gel, which is reflected in its higher elastic modulus (*G*′ ∼103 Pa and *G*′′ ∼102 Pa after two weeks of aging; Fig. S1†). The trends of *G*′ and *G*′′ values are parallel to those of gel formation efficiency and MGCs, *i.e.*, KLVF(I)F > KLVF(Br)F > KLVF(I)F(I). In particular, the latter yielded the weakest gel, which turned stiff enough for rheological measurements only after 2 weeks of aging. All other peptides, *i.e.*, WT, KLVFF(I), KLVFF(Br), KLVF(Br)F(Br) and KLVF(Cl)F(Cl), did not form gels in the same experimental set-up and all except KLVF(Br)F(Br) afforded colorless solutions. KLVF(Br)F(Br), instead, formed a homogeneously opaque, cloudy solution.

Interestingly, the pairs KLVF(I)F–KLVFF(I) and KLVF(Br)F–KLVFF(Br) are constitutional isomers, which show dramatically different macroscopic behaviors (gels *vs.* solutions), highlighting the specific role of the position of the halogen atom in the peptide sequence. Furthermore, the different behaviors of KLVF(I)F(I) compared to KLVF(Br)F(Br) also highlights a potential role of halogen atom polarizability in the self-assembly process. Hydrophobic interactions should not play a major role in determining the observed 15 mM-solution behaviors, *e.g.*, hydrogel formation,^24^ because mono-halogenated peptides have similar hydrophobicity, and the bis-brominated derivative is more hydrophobic than the mono-halogenated ones (Table S3†). Also, electrostatic interactions cannot fully explain the differences between the studied peptides.

The cloudy solution obtained from the KLVF(Br)F(Br) peptide was analyzed by using Polarized Optical Microscopy (POM) at room temperature. Birefringent textures in the form of spherulites were observed after 48 hours of preparation (Fig. S2†).

In order to investigate whether different solution behaviors are related to different morphologies of the halogenated peptide assemblies, imaging was performed using Transmission Electron Microscopy (TEM; 15 mM, 48 h after preparation) (Fig. 3).

**Fig. 3:**
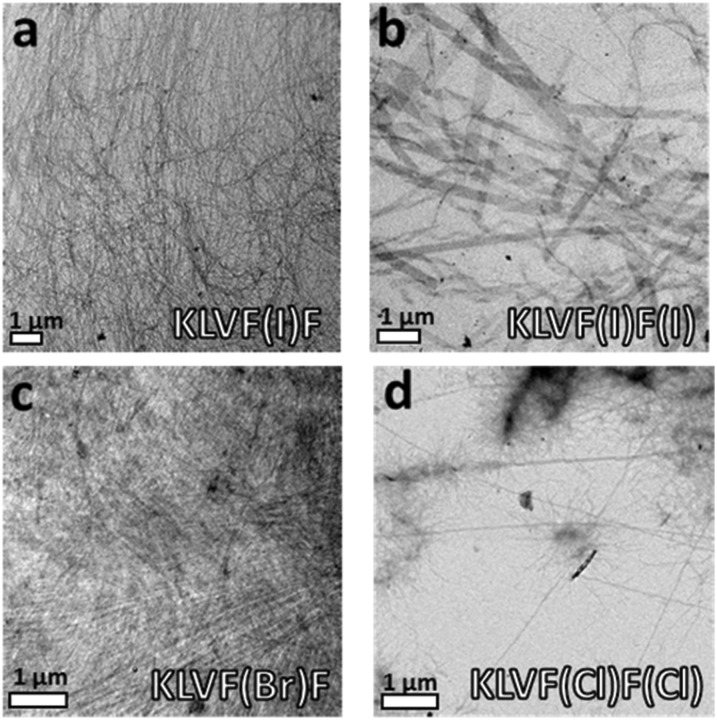
TEM images of the 15 mM dried gels/solutions of the halogenated derivatives of KLVFF showing different architectures upon varying the position, number, and nature of the halogen atoms.

As expected, the peptides forming the strongest gels – KLVF(I)F and KLVF(Br)F – showed a dense network of entangled fibrils (Fig. 3a and c), which is rather usual for amyloidal hydrogels. The weakest gel-forming peptide KLVF(I)F(I), instead, formed an entangled network of ribbon-like fibers, either straight or twisted, which may explain the cloudy appearance of the gel due to light scattering (Fig. 3b). A few long, thin, and straight fibrils were also observed in the case of KLVF(Cl)F(Cl), which, however, do not lead to the formation of the dense and entangled network needed for gel formation (Fig. 3d).

The non-gel-forming peptides KLVFF(I) and KLVF(Br)F(Br) showed the most interesting morphologies. In particular, spherical nanoparticles (NPs) were observed for the former (Fig. S3†), while spherical structures (∼300 nm) having a fuzzy, hairy interface, here referred to as “cotton balls”, were observed for the latter in the freshly prepared samples (Fig. 4). These aggregates are likely to act as nucleation centers for the undeveloped fibril structures branching out, but do not develop into a suitable hydrogel network. Derivative KLVFF(Br) and WT peptides, instead, formed only amorphous aggregates (Fig. S3†). Overall, the obtained TEM data clearly indicated the possibility of engineering different amyloid fibril architectures by changing the position, number, and nature of the halogen atoms in the peptide fragment KLVFF. Specifically, by placing a halogen atom on the inner phenylalanine residue of the sequence, an amyloid hydrogel was obtained, while different discrete morphologies could have been achieved when the terminal phenylalanine was functionalized with a halogen atom.

**Fig. 4:**
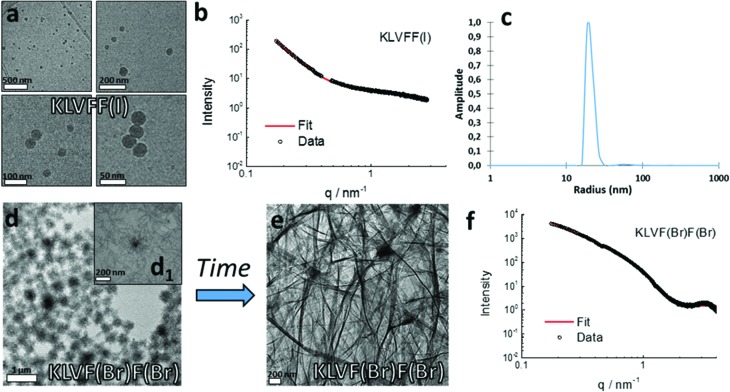
(a) Cryo-TEM images showing the spherical nanoparticles formed by KLVFF(I) 15 mM sample; (b) SAXS profile of 15 mM KLVFF(I) dispersion with fitting analysis according to a core–shell spherical form factor; (c) number average size distribution extracted from the DLS analysis of KLVFF(I) 5 mM dispersion; (d) TEM image of the 15 mM dried sample of KLVF(Br)F(Br) aged for 48 h; (d1) magnification of an isolated “cotton ball” structure formed by KLVF(Br)F(Br) from 15 mM solution aged for a week; (e) Cryo-TEM image showing an entangled fibrillar network formed by a one-month aged KLVF(Br)F(Br) 15 mM sample; (f) SAXS profile of KLVF(Br)F(Br) 15 mM samples with fitting analysis according to a bilayer form factor.

Due to their interesting morphologies and to ascertain whether the observed peptide nanostructures may be related to drying effects, the vitrified 15 mM aqueous solutions of KLVFF(I) and KLVF(Br)F(Br) were further investigated by Cryogenic-TEM (Cryo-TEM). As shown in Fig. 4a, the presence of the spherical NPs observed in the dried state was confirmed for the sample KLVFF(I). Imaged NPs (around 50 nm) showed smoother interfaces and more uniform shape when compared to NPs imaged in the dried state, indicating a possible NP agglomeration during drying.

A 5 mM water dispersion of KLVFF(I) was also studied by multi-angle DLS analysis giving bimodal auto-correlation functions composed of two populations characterized by 50 nm and 330 nm hydrodynamic radii, respectively (Fig. S4†). However, the number-averaged size distribution obtained for the same sample showed that the small population is much more abundant than the larger one, which is in agreement with the microscopy results (Fig. 4c).

On the other hand, the cryo-TEM imaging of individual “cotton balls” formed by the freshly prepared samples of KLVF(Br)F(Br) remained a challenge due to their relatively large size and compression upon cryo-vitrification. This leads to a uniform film formation without any contrast difference between the vitrified ice and the sample (Fig. S6†).^25^ However, the cryo-TEM analysis of one-month aged solutions indicated the presence of an entangled network of fibers with well visible nucleation points (Fig. 4e), suggesting that the “cotton balls” were evolved in a more thermodynamically stable architecture such as the typical amyloid fibril network. This structural transition is also observable macroscopically, since the freshly prepared 15 mM peptide solutions become more and more viscous over time until they form a gel (Fig. S7†).

The bulk characterization of one-month aged 15 mM peptide samples was carried out by Small Angle X-ray Scattering (SAXS) (see the ESI†). Halogenated derivatives showed a clearly different scattering behavior compared to KLVFF. The Bragg peaks at *q* = 1.5 nm^–1^ can be seen for samples KLVF(Br)F(Br) (Fig. 4f) and KLVF(I)F(I) (Fig. S12†), indicating the presence of periodic structures with around 2 nm spacing. The scattering patterns for these two samples were fitted using a bilayer model, previously employed for tape/ribbon-like structures.^26^ The obtained values indicate a bilayer thickness of 1.4 nm for the bis-brominated peptide and 0.9 nm for the bis-iodinated one. The KLVFF(I) scattering curve was fitted by a core–shell spherical model (Fig. 4b). The fitting yielded a diameter of 19.1 nm, which is in good agreement with the size determined by cryo-TEM. A long cylindrical shell model was, instead, used to analyze the KLVFF(Br) scattering curve (Fig. S13†). The fitting indicated a cylinder diameter of 5.7 nm.

We have recently demonstrated that iodination, alongside facilitating the phase determination, may be developed as a routine strategy to obtain the single-crystal X-ray structures of peptide segments otherwise difficult to crystallize. Thanks to this strategy, we have, for example, shed light on the elusive aromatic–aromatic interactions occurring in peptide segments containing phenylalanine, such as DFNKF.^27^ With the objective of determining whether noncovalent interactions involving halogen atoms may play a role in stabilizing the nanostructures observed in the present studies, we attempted the crystallization of the KLVF(I)F(I) derivative. Small, weakly diffracting, and poorly ordered crystals were obtained after two months upon the slow evaporation of a water/hexafluoro-2-propanol 9 : 1 mixture (see the ESI†). However, accurate structure solution was possible by using synchrotron radiation.

Similar to all other amyloid structures, the stacking of β-sheet pairs at the dry interface is the stable structural unit of the “cross-β-spine”,^28^ along which tightly interacting side chains form the self-complementary motif called “steric zipper”,^29^ which explains how very short peptide sequences are able to form such extended structures like amyloid fibrils. Interestingly, the single-crystal structure of KLVF(I)F(I) at 1.24 Å resolution shows the occurrence of intermolecular C=O···I contacts, *i.e.*, halogen bonds, between the peptides belonging to the adjacent β-sheets (Fig. 5a). The two asymmetric O···I distances are O_1···I_5 3.34(3) Å, and O_2···I_4 3.43(3) Å, with C=O···I and O···I–C angles of 111(2)° and 103(2)°, and 165(1)° and 159(1)°, respectively. Importantly, the O_2···I_4 halogen bonding occurs orthogonally^30^ to the O_2···N_3 hydrogen bond of the β-sheet (N···O distance 2.99(3) Å; N···O···I angle 88.5(8)°) (Fig. 5b). This is the first structural evidence of halogen bonding stabilizing the steric zipper of amyloidogenic peptides. The occurrence of halogen bonding causes KLVF(I)F(I) monomers to pair in parallel mode, while in the structure of the non-iodinated sequence KLVFFA,^31^ monomers pair in antiparallel mode, resulting in a different type of “steric zipper” (Fig. S15†). This result confirms the possibility to exploit the halogen bond as a tool to engineer the β-sheet and the self-assembly of amyloidal peptides.^17^ The single-crystal structures of KLVF(Br)F(Br) and KLVF(Cl)F(Cl) were also successfully determined. The former is isostructural with KLVF(I)F(I) and shows a weaker halogen bonding, due to the lower polarizability of Br, while the latter shows no sign of halogen bonding as can be expected from a chlorobenzene derivative (see the ESI† for the detailed structural analysis of KLVF(Br)F(Br) and KLVF(Cl)F(Cl)).^19^

**Fig. 5:**
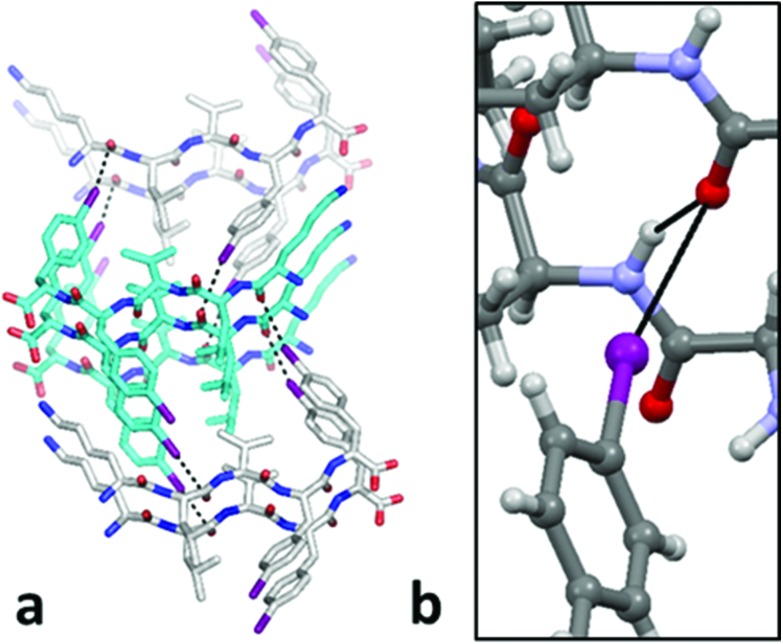
(a) “Steric zipper” class 4 motif^26^ formed by KLVF(I)F(I), showing the intermolecular halogen bonds between the iodine atom and the carbonyl oxygen belonging to the adjacent β-sheets. (b) Orthogonality between the halogen bond and the hydrogen bond in the crystal structure of KLVF(I)F(I). Color code: C, grey and cyan; O, red; N, blue and violet; I, magenta; H, white. Halogen and hydrogen bonds as solid black lines. Solvent molecules are omitted for clarity.

Finally, very interesting insights into a possible mechanism correlating a nanostructure architecture and the role of the halogens in the self-assembly process came from a detailed analysis of the IR spectra of all peptides in 48 h-aged 15 mM solutions. Interestingly, the spectra of KLVF(I)F and KLVF(Br)F peptides clearly show the vibration of the β-sheet carbonyl bond at around 1630 cm^–1^. This band is even more intense and red-shifted in the KLVF(I)F(I) derivative (Fig. S23†). This may be explained by the extra β-sheet stabilization offered by the occurrence of the halogen bonds detected in its X-ray structure. The presence of the β-sheet in these three derivatives is perfectly compatible with the formation of amyloid fibrils and ribbons in the solution. On the other hand, no clear IR signature of the β-sheet could be detected in the IR spectra of all other peptides. This, again, is perfectly compatible with the formation of spherical nanostructures. In our hypothesis, when the halogenated residue is at the C-terminus it is not involved in the formation of halogen bonds, as also observed in another system.^27^ Conversely, when the halogen residue is in a more internal position of the peptide sequence, it forms halogen bonds with the carbonyl oxygen of a nearby peptide molecule, resulting in a stronger β-sheet formation. A synergistic effect can, instead, operate in the case of the bis-iodinated derivative, where both iodine atoms are simultaneously involved in the formation of halogen bonds with carbonyl oxygens,^32^ behaviour nicely displayed in the reported crystal structure (Fig. 5b). The robustness of the C=O···I supramolecular synthon suggests its possible key role in dictating the architectures of the observed peptide nanostructures. More detailed investigation on this mechanism will be reported in a separate research article.

## Conclusions

Herein we have reported that single-atom hydrogen-for-halogen replacement at the *p*-position of either one or both Phe benzene rings expands the structural landscape of KLVFF. At least four solution-stable polymorphic architectures have been obtained comprising fibrils, ribbons, nanoparticles, and “cotton balls”, which are not shown by the WT peptide under the same experimental conditions. The position, nature, and number of the introduced halogen atoms dictate the specific formation of each determined architecture. The present hypothesis of a key structural role played by the halogen bond is corroborated by crystallographic determinations that fully demonstrate the potential that the halogen bond has to engineer peptide self-assembly.

## Experimental

### Rheology

Rheology experiments were performed using a TA instrument ARG2 Rheometer. A 20 mm stainless steel, parallel-plate geometry was used with a gap distance of 1000 μm. Oscillatory frequency sweep studies were performed in a range of 0.1–100 rad s^–1^, using a 0.5% strain. Oscillatory amplitude sweep studies were conducted from 0.01 to 100% strain with an angular frequency of 1 rad s^–1^. The ring cast method was used for hydrogel preparation at a peptide concentration of 15 mM. All measurements were repeated a minimum of three times.

### Dynamic light scattering

Dynamic light scattering measurements were performed on an ALV/CGS-3 Platform-based Goniometer System equipped with an ALV-7004 correlator and an ALV/CGS-3 goniometer. The signal was detected by using an ALV-Static and Dynamic Enhancer detection unit. The light source was the second harmonic of a diode-pumped Coherent Innova Nd:YAG laser (*λ* = 532 nm), linearly polarized in the vertical direction. Measurements were performed at 25 °C. Approximately 1 mL of the sample solution was transferred into the cylindrical Hellma scattering cell.

### Transmission electron microscopy

TEM bright field images were acquired using a Philips CM200 electron microscope operating at 200 kV equipped with a field emission gun filament. A Gatan US 1000 CCD camera was used and 2048 × 2048 pixel images with 256 grey levels were recorded. The suspension was dropped onto a 200 mesh carbon-coated copper grid and air dried for several hours before analysis. All the samples were visualized without the negative staining procedure.

### Cryo-TEM

The cryo-TEM images were collected using a JEM 3200FSC field emission microscope (JEOL) operated at 300 kV in bright field mode with an Omega-type Zero-loss energy filter. The images were acquired with a Gatan digital micrograph software while the specimen temperature was maintained at –187 °C. The Cryo-TEM samples were prepared by placing a 3 μL aqueous dispersion of nanoparticles/clusters on a 200 mesh copper grid with a holey carbon support film (CF-Quantifoil) and were plunge freezed using Vitrobot with 2 s blotting time under a 100% humidity.

### Small angle X-ray scattering

Solution SAXS measurements were performed on the bioSAXS beamline BM29 at the ESRF, Grenoble, France. Solutions (1 wt%) were loaded in the PCR tubes in an automated sample changer. SAXS data were collected using a Pilatus 1 M detector. The sample–detector distance was 2.84 m. The X-ray wavelength was 0.99 Å.

### Single crystal X-ray diffraction

KLVF(I)F(I), KLVF(Br)F(Br) and KLVF(Cl)F(Cl) crystals were obtained as solvated species by dissolving the peptide in a water/hexafluoro-2-propanol 90 : 10 mixture. Crystals suitable for XRD analysis were obtained after two months of slow evaporation. Data collections were performed at the X-ray diffraction beamline (XRD1) of the Elettra Synchrotron, Trieste (Italy). The crystals were dipped in perfluoropolyether vacuum oil (Fomblin) and mounted on the goniometer head with a nylon loop. Complete datasets were collected at 100 K (nitrogen stream supplied through an Oxford Cryostream 700) by the rotating crystal method. Data were acquired using a monochromatic wavelength of 0.850 Å for KLVF(I)F(I) and 0.700 Å for KLVF(Br)F(Br) and KLVF(Cl)F(Cl) on a Pilatus 2 M hybrid-pixel area detector.

Further details about the experimental set-up, data analysis and peptide synthesis are available in the ESI.†

CCDC 1454960, 1454959, and 1494096 contain the supplementary crystallographic data for this paper.

## Author contributions statement

A. P. performed most of the peptide self-assembly and crystallization studies. C. P. and N. performed the cryo-TEM analysis. A. G. synthesized the peptides. N. D. performed the single crystal X-ray analysis and refined the crystal structures. V. C. and I. W. H. performed the SAXS experiments. P. M., O. I., G. T. and F. B. conceived the experiments and contributed to the discussion of the results. All authors have given approval to the final version of the manuscript.

## Competing financial interest

The authors declare no competing financial interest.

## Supplementary Material

Supplementary information
