# Supplementary material for: Halogenation dictates the architecture of amyloid peptide nanostructures
Source: Nanoscale. 2017 Jun 22;9(28):9805–10. doi: 10.1039/c7nr03263c (PMC5708343; doi:10.1039/c7nr03263c)
Supplement: Supplementary file 1 [file NR-009-C7NR03263C-s001.pdf]

Electronic Supplementary Material (ESI) for Nanoscale.
This journal is © The Royal Society of Chemistry 2017

Halogenation Dictates Architecture of Amyloid Peptide Nanostructures

Andrea Pizzi,[a] Claudia Pigliacelli,[b] Alessandro Gori,[c] Nonappa,[b] Olli Ikkala,[b] Nicola Demitri,[d] Giancarlo Terraneo,[a] Valeria Castelletto,[e] Ian W. Hamley,[e] Francesca Baldelli Bombelli,[a] Pierangelo Metrangolo*[a,b,c]

[a] Laboratory of Supramolecular and BioNano Materials (SupraBioNanoLab), Department of Chemistry, Materials, and Chemical Engineering "Giulio Natta", Politecnico di Milano, Via Luigi Mancinelli 7, Milano I-20131, Italy

[b] Department of Applied Physics, Aalto University, Espoo, FI-02150, Finland

[c] Istituto di Chimica del Riconoscimento Molecolare – National Research Council of Italy (ICRM-CNR), Via Mario Bianco 9, 20131 Milano, Italy

[d] Elettra – Sincrotrone Trieste, S.S. 14 Km 163.5 in Area Science Park, 34149 Basovizza – Trieste, Italy

[e] Department of Chemistry, University of Reading, Whiteknights, Reading, RG6 6AD, UK

Supplementary information

Supplementary information content

1) Materials

2) Methods

2.1) Hydrogel Preparation

2.2) Rheology

2.3) Polarized Optical Microscope (POM) analysis

2.4) Transmission Electron Microscopy (TEM)

2.5) Dynamic Light Scattering (DLS).

2.6) Cryogenic Transmission Electron Microscopy (cryo-TEM).

2.7) Small Angle X-ray Scattering (SAXS)

2.8) X-ray diffraction analysis

2.9) Infrared spectroscopy (FTIR)

2.10) Circular Dichroism (CD) Spectroscopy

2.11) Confocal Microscopy

2.12) Congo Red Staining

2.13) Peptide Synthesis

2.14) Tables

3) References

1) Materials

Congo red, Rhodamine B, NaCl, Phosphate buffered saline and D_2O were purchased from Sigma-Aldrich and used without further purification. Peptides with confirmed amino acid analysis (purity ≥ 98%), were purchased from Biopeptek (Malvern, USA). The integrity of all peptides was confirmed by ion spray mass spectrometry and the purity was determined by reverse phase high-pressure liquid chromatography (HPLC).

The same peptides were also synthetized in house through classical solid-phase protocols. CTC resin and N-α-Fmoc-L-amino acids used during chain assembly were purchased from Iris Biotech GmbH (Marktredwitz, Germany). Ethyl cyanoglyoxylate-2-oxime (Oxyma) was purchased from Novabiochem (Darmstadt, Germany), N,N'-dimethylformamide (DMF) and trifluoroacetic acid (TFA) were from Carlo Erba (Rodano, Italy). N,N'-diisopropylcarbodiimide (DIC), dichloromethane (DCM) and all other organic reagents and solvents, unless stated otherwise, were purchased in high purity from Sigma-Aldrich (Steinheim, Germany). All solvents for solid-phase peptide synthesis (SPPS) were used without further purification. HPLC grade acetonitrile (ACN) and ultrapure 18.2 Ω water (Millipore-MilliQ) were used for the preparation of all buffers for liquid chromatography. The chromatographic columns were from Phenomenex (Torrance CA, USA). HPLC eluent A: 97.5% H_2O, 2.5% ACN, 0.7%TFA; HPLC eluent B: 30% H2O, 70% ACN, 0.7%TFA

2) Methods

2.1 Hydrogel preparation

Hydrogels were prepared by dissolving each peptide (15 mM for halogenated peptides and 64 mM for the wild-type peptide KLVFF) in deionized water (18.2 MΩ.cm) or in D_2O (≥ 99.9 atom % deuterium). The glass vials containing the 500 µL solutions were sealed, sonicated for 20 seconds, heated using a heat gun until complete dissolution of the peptides, before slow cooling to r.t. All samples were stored at r.t. for 48 hours before analysis.

Peptide solutions were freshly prepared in deionized water (18.2 MΩ.cm), sonicated for 20 seconds, and gently warmed to reach 90 °C before filtration through a 0.22 µm Millipore filter. The peptide solutions were stored in sealed vials at r.t. for varying time points before analysis.

2.2 Rheology

Rheology experiments were performed using a TA instrument ARG2 Rheometer. A 20 mm Stainless steel, parallel-plate geometry was used with a gap distance of 1000 µm. Oscillatory frequency sweep studies were performed for a range of 0.1-100 rad/s, using a 0.5% strain. Oscillatory amplitude sweep studies were conducted from 0.01 to 100% strain with an angular frequency of 1 rad/s. The ring cast method was used for hydrogel preparation at a peptide concentration of 15 mM. The peptide solution was sonicated for 20 s in a sealed glass vial before heating to 90 °C to afford complete dissolution of the peptide. After cooling the solutions were transferred into ring-casts of 22 mm diameter and placed in tightly sealed tissue-culture dishes for 48 hours. All measurements were repeated a minimum of three times.

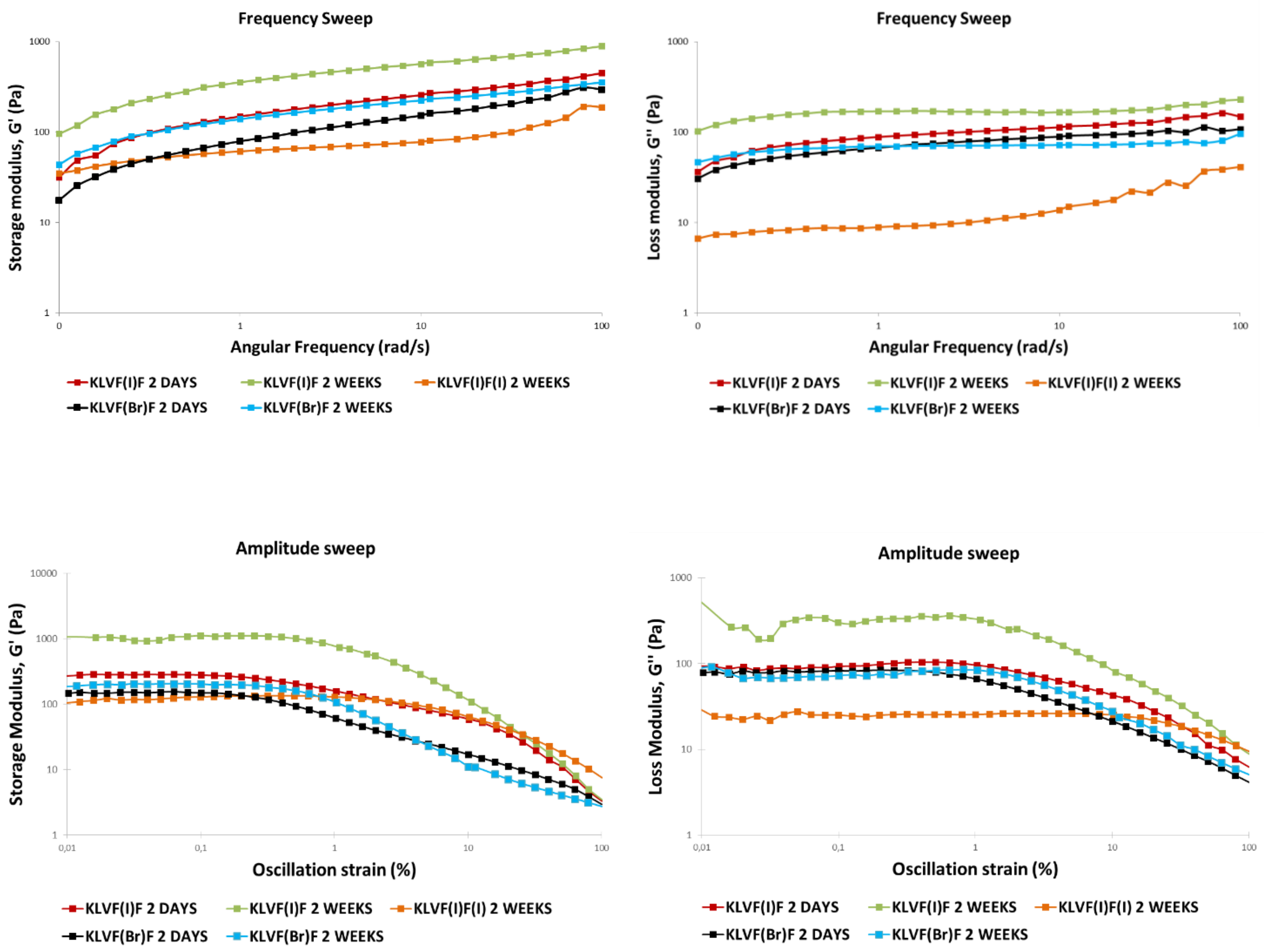

Figure S1. Reological characterization of the gels by frequency sweep studies whereby the storage modulus (G’) was recorded as a function of angular frequency (ω). Amplitude sweep studies of peptide hydrogels showing G’’ as a function of oscillation strain (γ).

2.3 Polarized Optical Microscope (POM) analysis

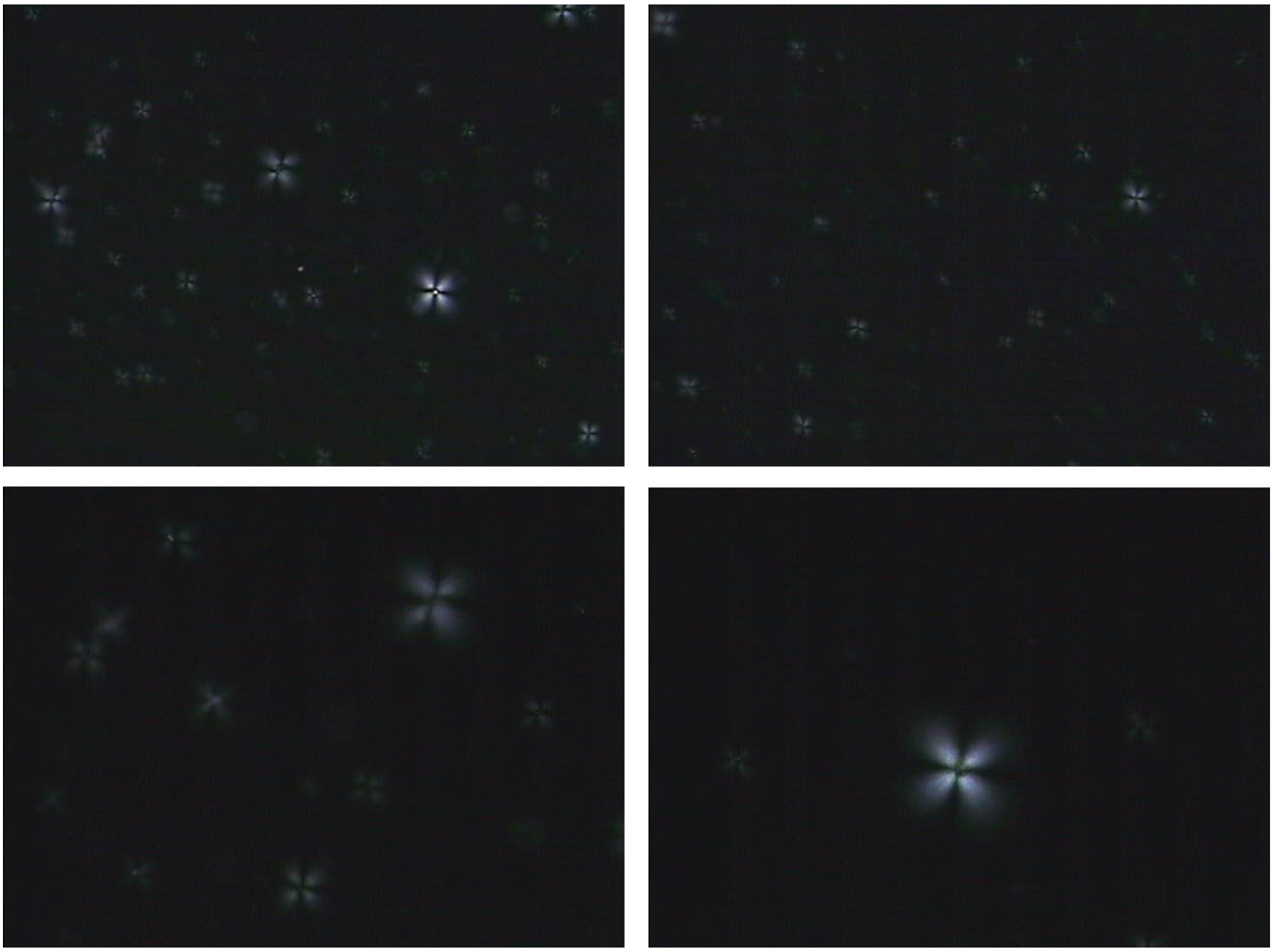

Figure S2. Polarized Optical Microscope images of a 48h aged KLVF(Br)F(Br) 15 mM solution, showing some birefringent spherulites. These images were registered with a Leica DM4500P POM system that was equipped with a Canon EOS 60D camera.

2.4 Transmission Electron Microscopy (TEM)

TEM bright field images were acquired using a Philips CM200 electron microscope operating at 200 kV equipped with a Field Emission Gun filament. A Gatan US 1000 CCD camera was used and 2048x2048 pixels images with 256 grey levels were recorded. The suspension were dropped onto a 200 mesh carbon-coated copper grid and air dried for several hours before analysis. No negative staining was used.

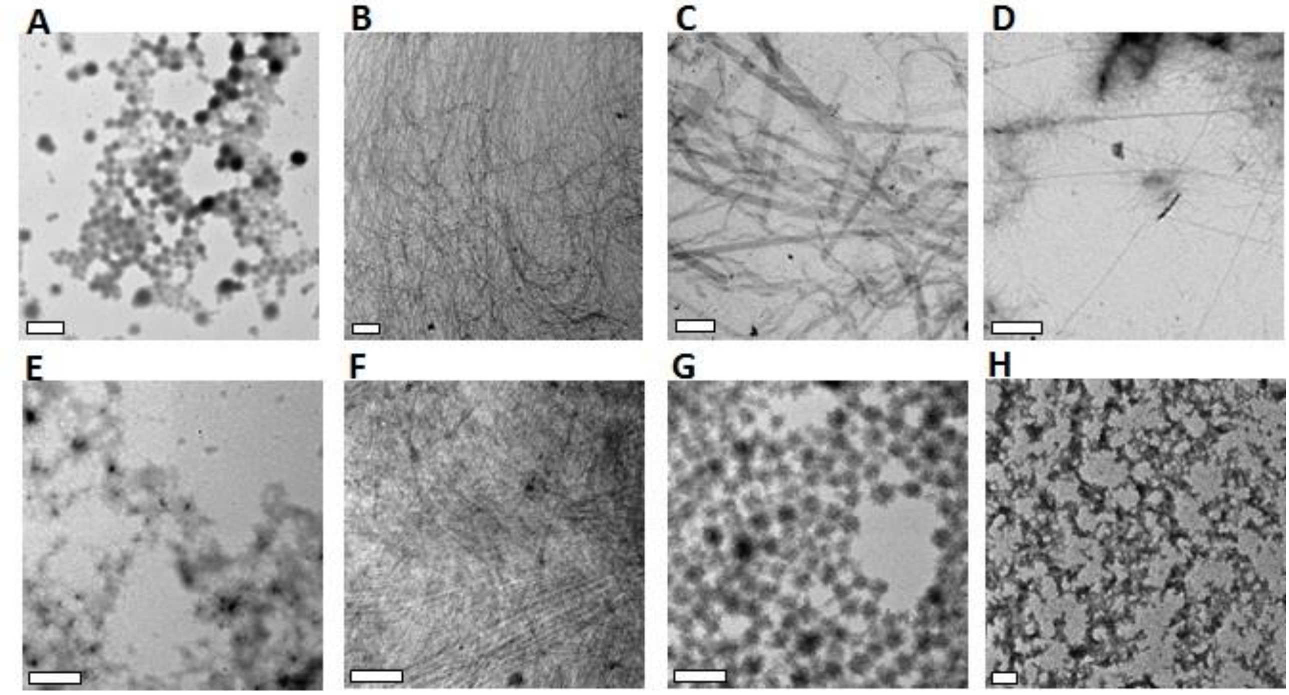

Figure S3. TEM images of 15 mM gels/solutions of the halogenated derivatives of KLVFF showing different self-assemblies varying position, number and nature of the halogen atoms. KLVFF(I) (A); KLVF(I)F (B); KLVF(I)F(I) (C); KLVF(Cl)F(Cl) (D); KLVFF(Br) (E); KLVF(Br)F (F); KLVF(Br)F(Br) (G); KLVFF (H). The scale bar is 1µm.

2.5 Dynamic Light Scattering (DLS).

Dynamic Light Scattering measurements were performed on an ALV/CGS-3 Platform-based Goniometer System equipped with an ALV-7004 correlator and an ALV / CGS-3 goniometer. The signal was detected by an ALV-Static and Dynamic Enhancer detection unit. The light source was the second harmonic of a diode-pumped Coherent Innova Nd:YAG laser (λ = 532 nm), linearly polarized in the vertical direction. Measurements were performed at 25 °C. Approximately 1 mL of sample solution was transferred into the cylindrical Hellma scattering cell.

The dynamic information on particles present in the peptide solutions were derived from the normalized autocorrelation function $g_2(q, \tau)$ of the scattered intensity, which is measured according to

$$g_2(q,\tau) = \frac{\langle I(q,t)I(q,t+\tau)\rangle}{\langle I(q,t)\rangle^2}$$

where q is the scattering vector, τ is the relaxation time and I is the scattered intensity. Data analysis has been performed with two different methods: in the first one, the autocorrelation functions have been analyzed through Laplace inversion (CONTIN algorithm), which resulted in a double distribution of the decay rates associated to two distinct populations. In the second method, the same autocorrelation functions have been analyzed using a double exponential decay model, yielding two distinct decay rates. For each sample, at least three measurements were performed at four different angles (70°, 90°,110°,130°), corresponding to four different scattering vectors (q):

$$q = (4\pi n/\lambda)\, sin(\theta/2)$$

where n is the refractive index of the medium and θ is the scattering angle. The extracted decay rates were plotted versus the square scattering vectors (q^2) showing a linear dependence typical of Brownian motion. The slope of this curve represents the averaged translational diffusion coefficient (D_T), from which, through the Stokes-Einstein equation

$$R_H = k_B T/6\pi\eta D_T$$

(k_B is the Boltzmann constant, T is the temperature and η is the viscosity) it is possible to calculate the averaged hydrodynamic radius (R_H) associated to each population. This analysis, although neglecting polydispersity of the two populations, is a good control on the Laplace inversion method (CONTIN), which can produce artefacts if the autocorrelation functions are significantly noisy, with low amplitude to baseline ratio.

a

Aging	1 month	
Fitting model	Double exponential	
Scattering angles	70° 90° 110° 130°	
	Population I	Population II
D (cm^2 s^{-1})	$4.51 \cdot 10^{-8}$	$7.27 \cdot 10^{-9}$
R_H (nm)	53	330

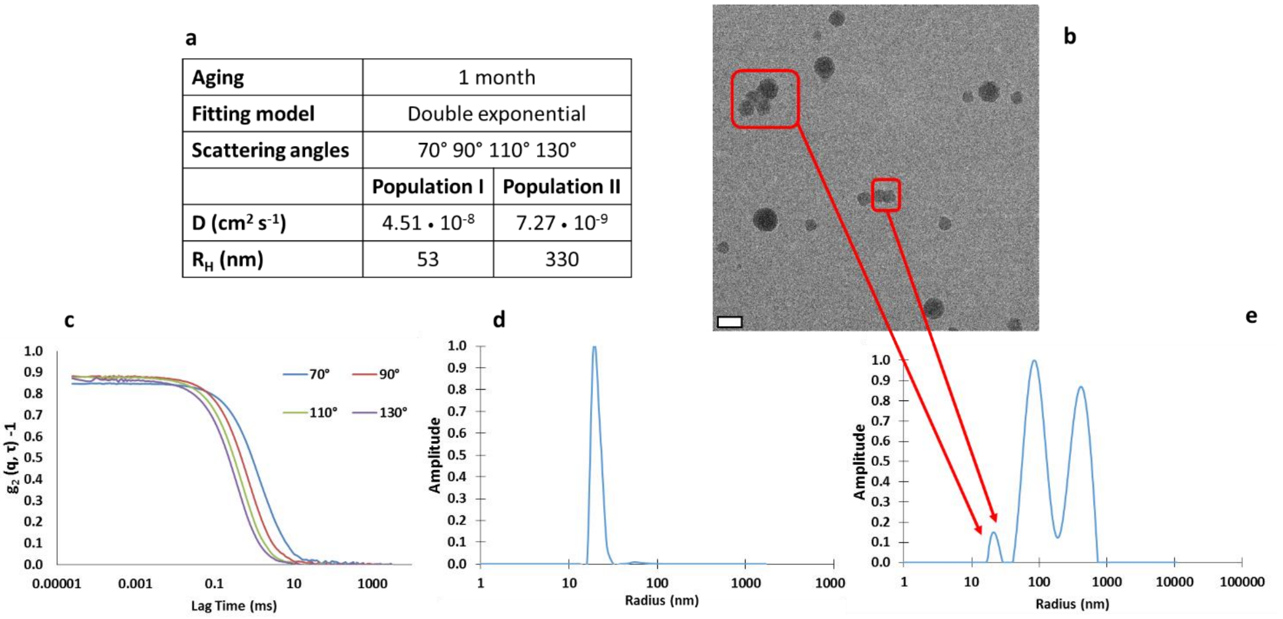

Figure S4. DLS analysis for KLVFF(I) 5 mM. Table summarizing experimental conditions and fitting results a) Cryo-TEM showing some NPs clusters. Scale bar 50 nm b) Autocorrelation functions associated to the analyzed scattering angles c) Number averaged distribution function calculated with CONTIN at 90° d) Intensity averaged distribution function calculated with CONTIN at 90° e).

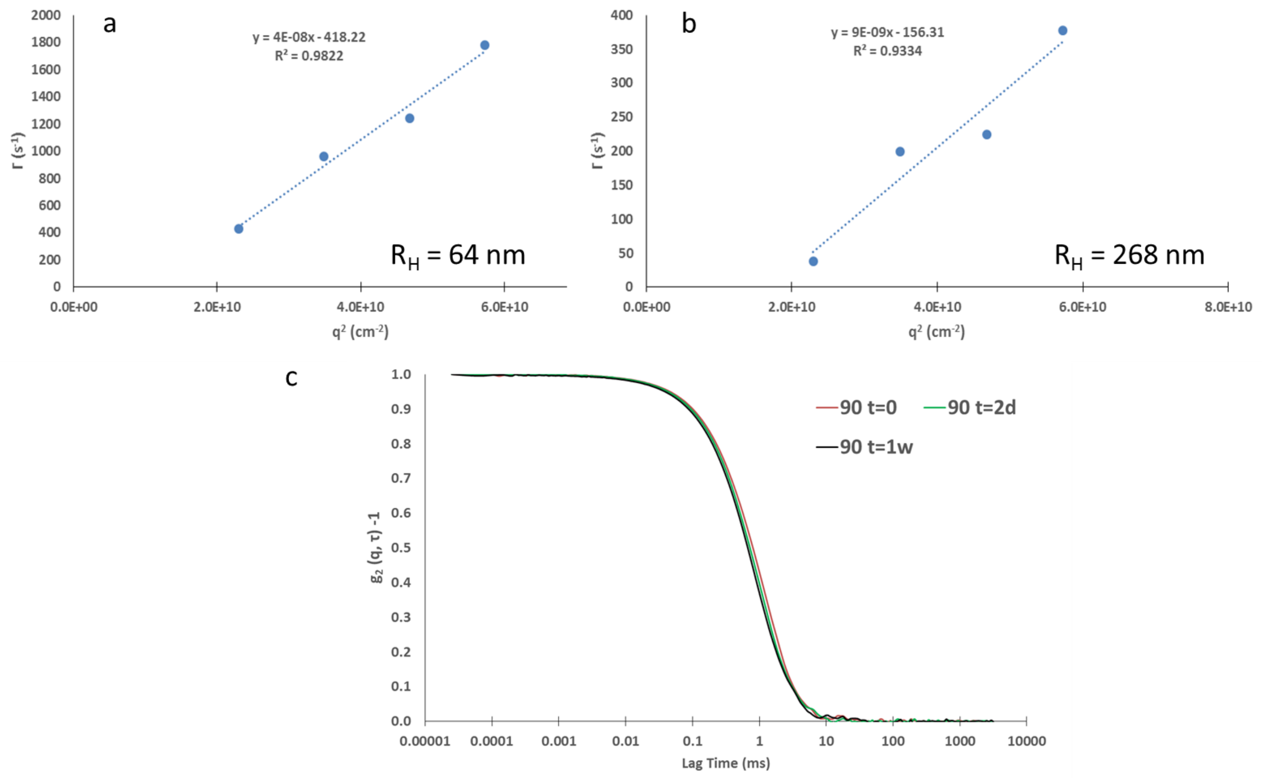

Figure S5. a) b) Multi angle analysis for KLVF(Br)F(Br) 5 mM solution after 1 week aging. The autocorrelation functions associated to four different angles (70°, 90°, 110°, 130°) were fitted with a double exponential model, leading to two different decay times, associated to two distinct populations. c) Evolution of the autocorrelation function at 90° for KLVF(Br)F(Br) 5 mM solution.

2.6 Cryogenic Transmission Electron Microscopy (cryo-TEM).

The cryo-TEM images were collected using JEM 3200FSC field emission microscope (JEOL) operated at 300 kV in bright field mode with Omega-type Zero-loss energy filter. The images were acquired with Gatan digital micrograph software while the specimen temperature was maintained at -187 ^{o}C. The Cryo-TEM samples were prepared by placing 3 µL aqueous dispersion of nanoparticles/clusters on a 200 mesh copper grid with holey carbon support film (CF-Quantifoil) and plunge freezed using vitrobot with 2s blotting time under 100% humidity. No negative staining was used.

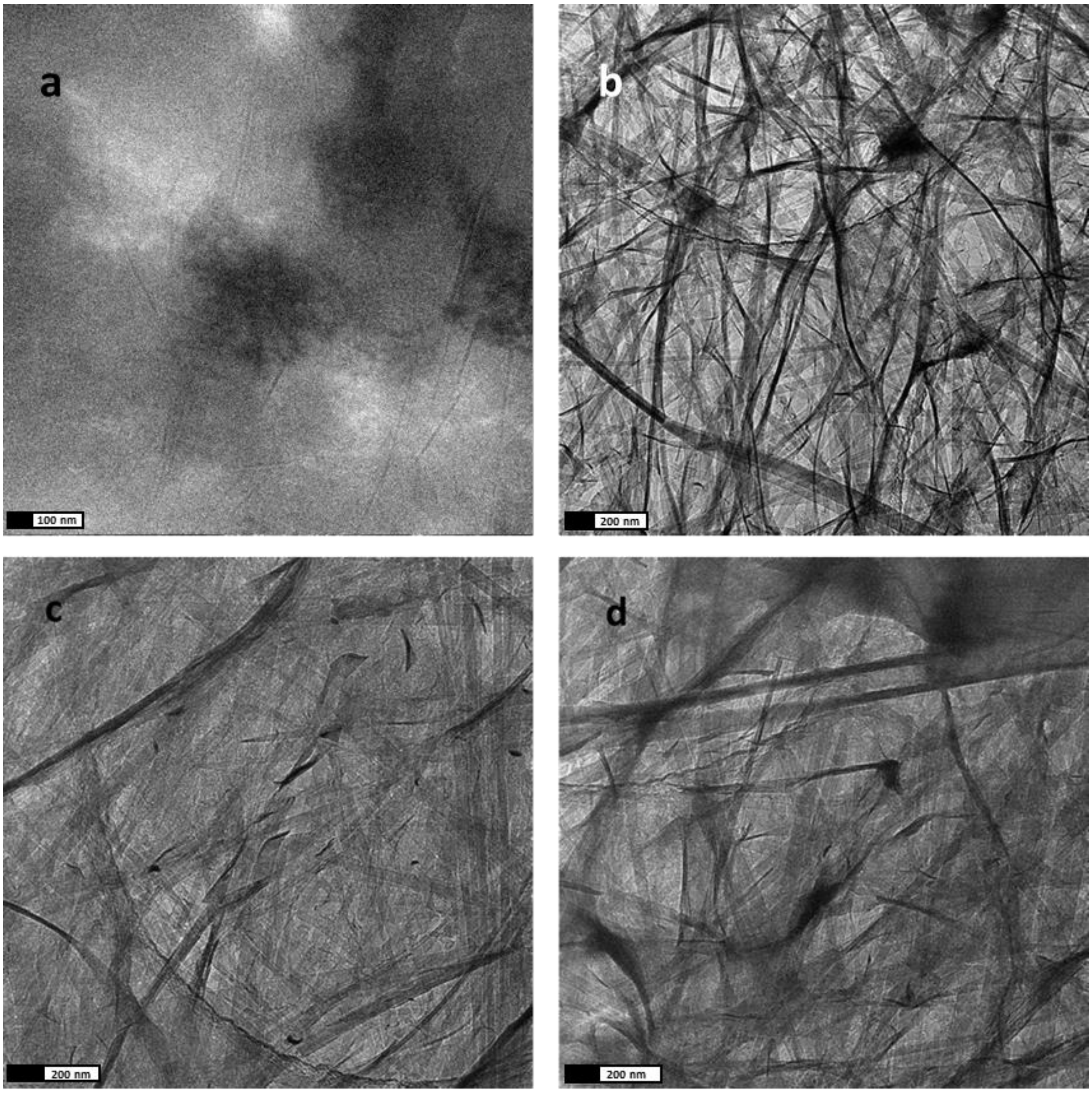

Figure S6. cryo-TEM images of KLVF(Br)F(Br) 15 mM. a) 48 h after sample preparation. b) c) d) one month after sample preparation.

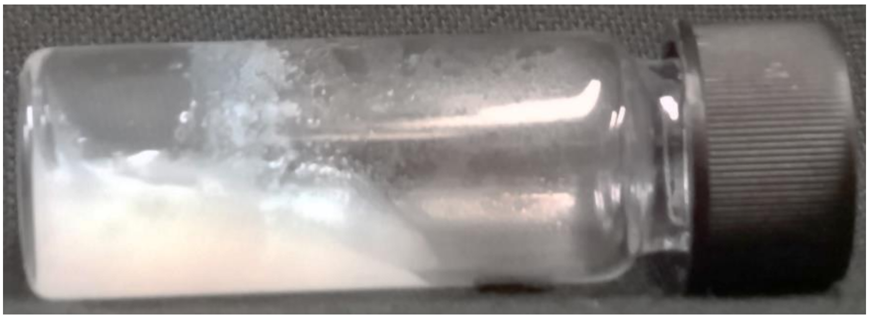

Figure S7. A KLVF(Br)F(Br) 15 mM sample after one month aging. The solution appears solid-like.

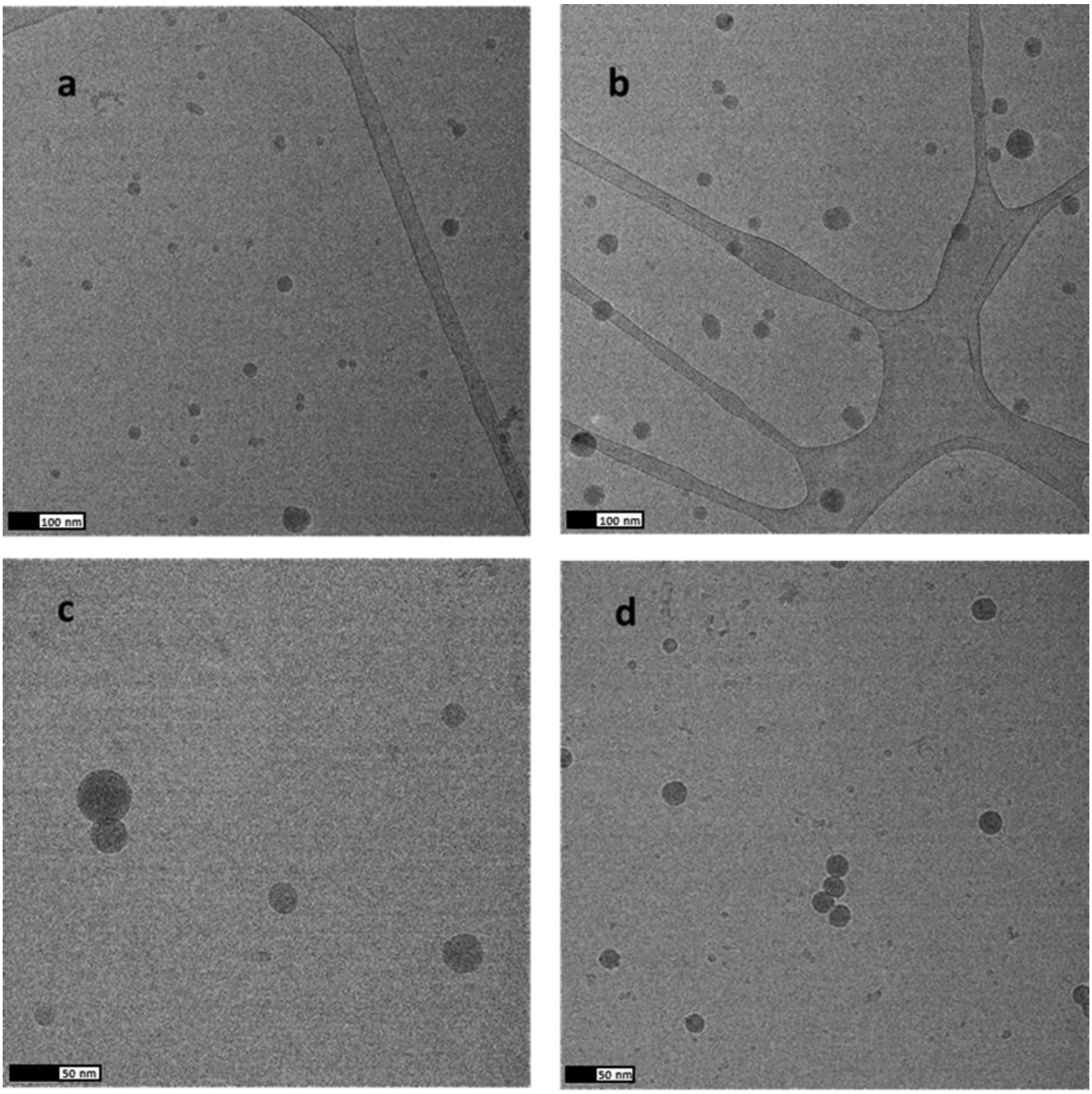

Figure S8. cryo-TEM images of **KLVFF(I)** 15 mM

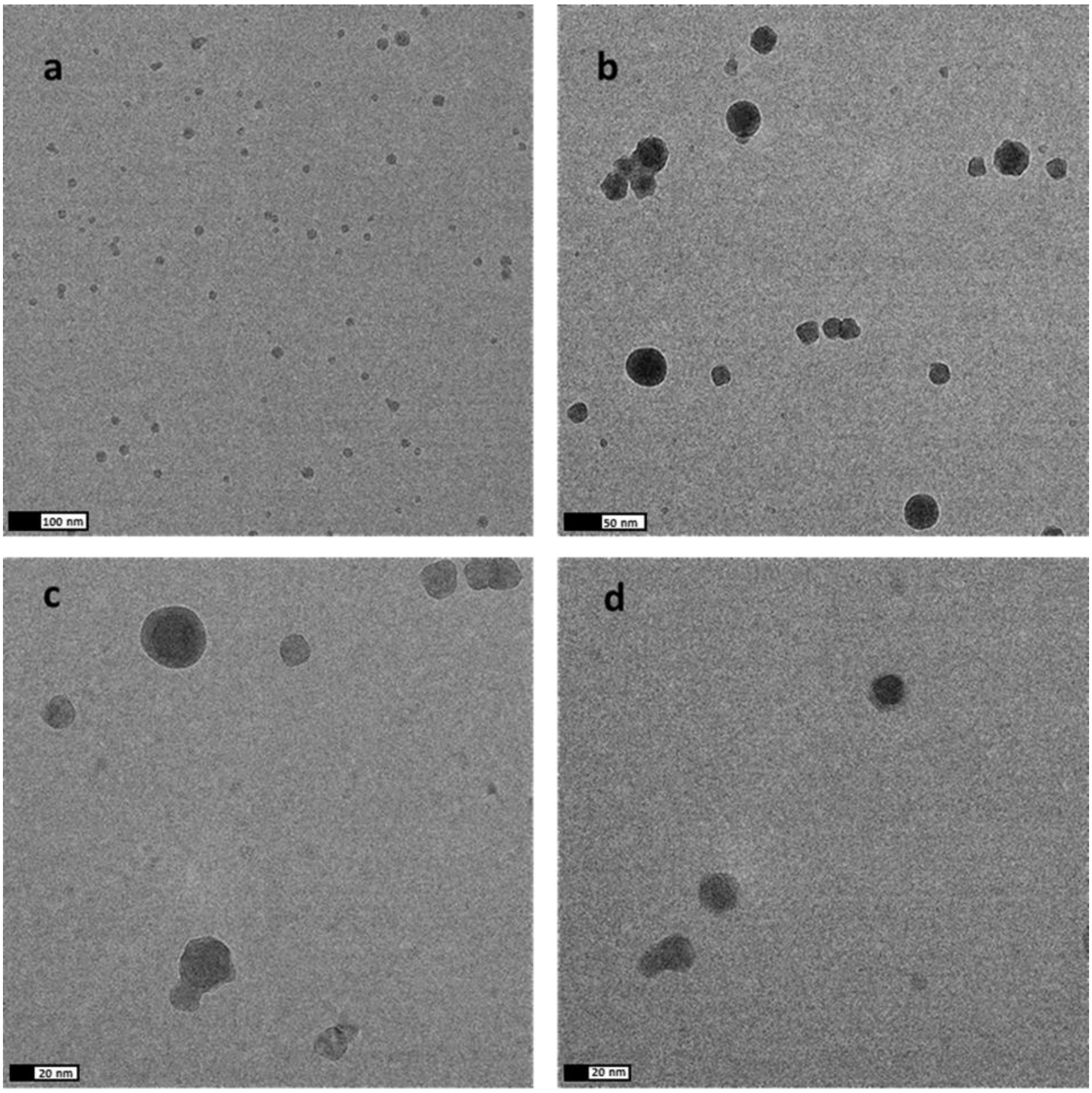

Figure S9. cryo-TEM images of **KLVFF(I)** 5 mM

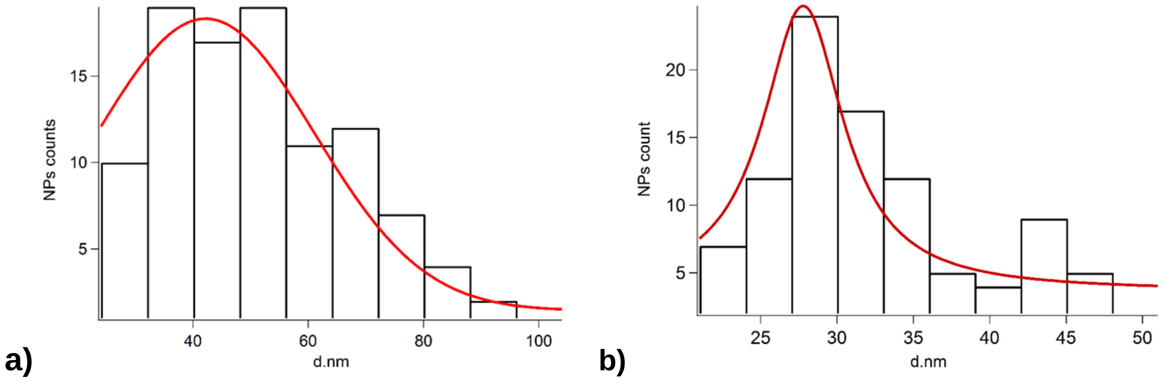

Figure S10. a) KLVFF(I) 15 mM size distribution obtained by Cryo-TEM. The average diameter is 42 ± 26 nm. b) KLVFF(I) 5 mM size distribution obtained by Cryo-TEM. The average diameter is 27 ± 10 nm. The red line represents the Lorentzian fitting of the size distribution histogram.

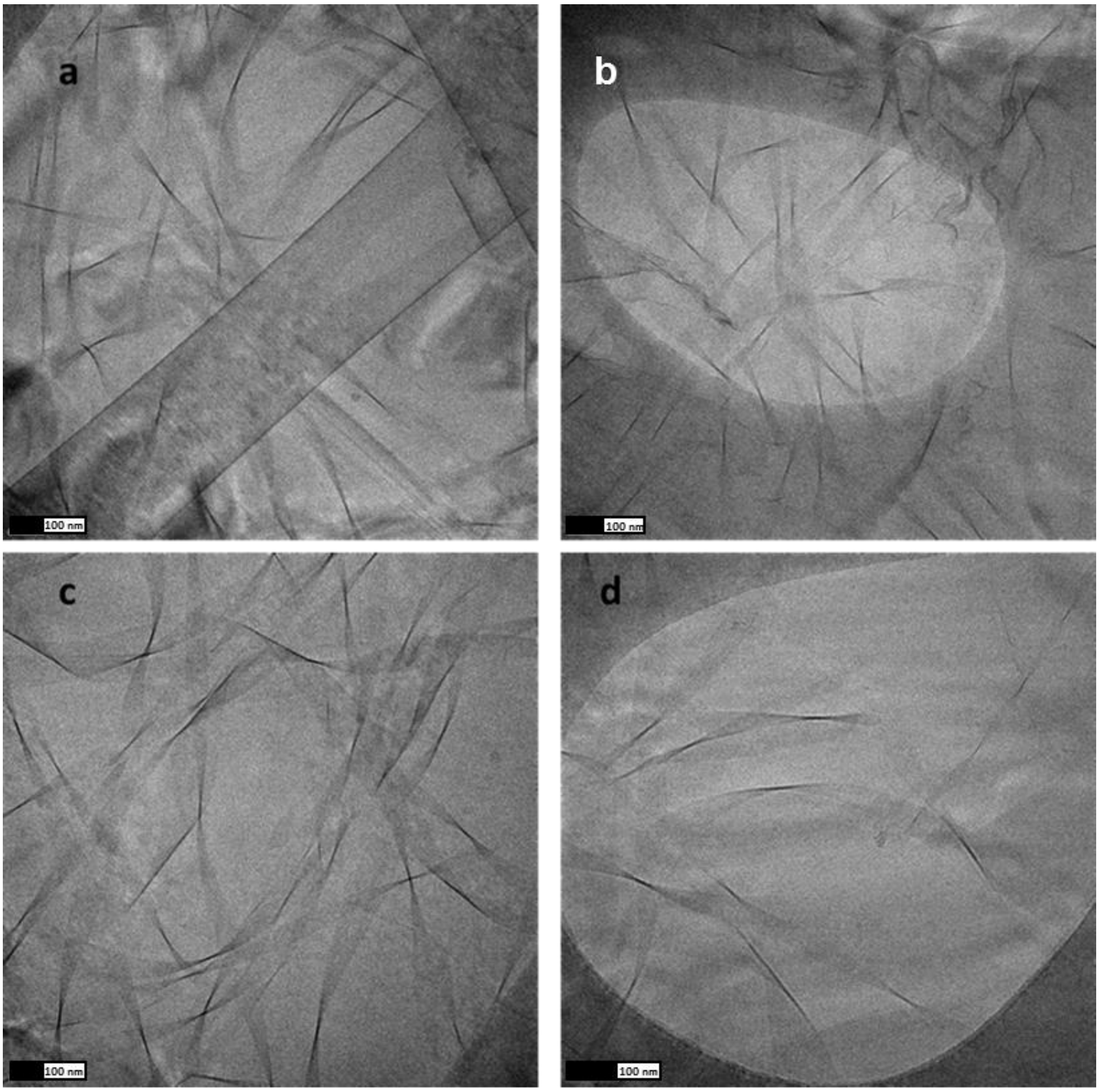

Figure S11. Cryo-TEM images of KLVF(I)F(I) 15 mM samples aged for two months

2.7 Small Angle X-ray Scattering (SAXS)

Solution SAXS measurements was performed on the bioSAXS beamline BM29 at the ESRF, Grenoble, France. Solutions (1 % wt) were loaded in PCR tubes in an automated sample changer. SAXS data was collected using a Pilatus 1 M detector. The sample−detector distance was 2.84 m. The X-ray wavelength was 0.99 Å.

SAXS Data were fitted using the software SASfit[1S]

Data for KLVFF(I) were fitted to a spherical shell model with outer radius R_{out} = 14.6 nm (Gaussian polydispersity width ΔR_{out} = 8.9 nm), the inner radius was R_{in} = 1.39 nm, the scattering density was η = 0.0027 (arbitrary units). A constant background BG = 2 was included in the fit.

Data for KLVF(Br)F(Br) were fitted to a Gaussian bilayer model[S2] (used to describe tape structures for peptide assemblies in recent papers by the Hamley group e.g. ref.S3). The fit parameters were an overall scale factor s = 0.044, bilayer thickness 1.39 nm with Gaussian polydispersity Δt = 0.33 nm, width of inner Gaussian σ_{in} = 1.10nm, inner Gaussian scattering density η = -0.011 (arb. units), outer Gaussian width σ_{out} = 0.88 nm (arb. units) and outer Gaussian scattering density η = 0.0187 (arb. units). A constant background BG =1.479 was included in the fit.

Data for KLVFF(Br) were fitted with a long cylindrical shell model. The fit parameters: overall scale factor σ=0.0032, radius R =5.71 with Gaussian polydispersity ΔR=3.24, shell thickness t_s=0.81 nm, cylinder length L=500 nm, scattering density of core η_c=0.021 (arb. units), scattering density of shell η_s=-0.18 (arb. units). A constant background BG = 0.797 was included in the fit. There is a broad peak in the SAXS data near q =0.04 nm-1 which corresponds to a structure factor peak due to inter-fibril correlations which was not included in the form factor fitting.

Data for KLVF(I)F(I) were fitted to a Gaussian bilayer model[S2] (used to describe tape structures for peptide assemblies in recent papers by the Hamley group e.g. ref.S3). The fit parameters were an overall scale factor s = 0.037, bilayer thickness 0.87 nm with Gaussian polydispersity Δt = 0.13 nm, width of inner Gaussian σ_{in} = 0.952 nm, inner Gaussian scattering density η = 0.02 (arb. units), outer Gaussian width σ_{out} = 1.77 nm (arb. units) and outer Gaussian scattering density η = 0.0034 (arb. units). A constant background BG =1.33 was included in the fit.

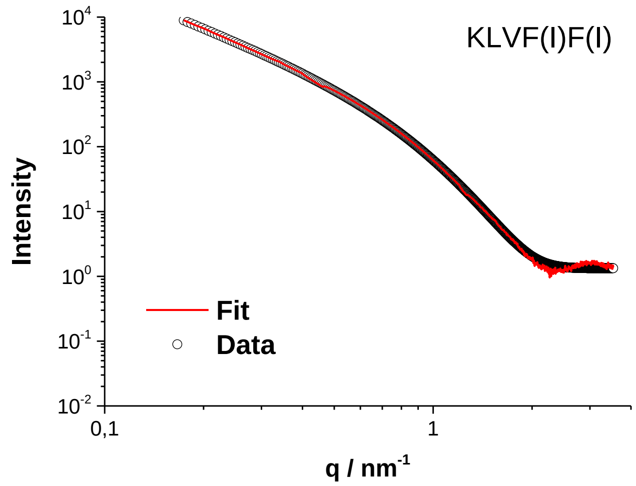

Figure S12. SAXS profile of 15 mM KLVF(I)F(I) hydrogel with fitting analysis according to a bilayer model.

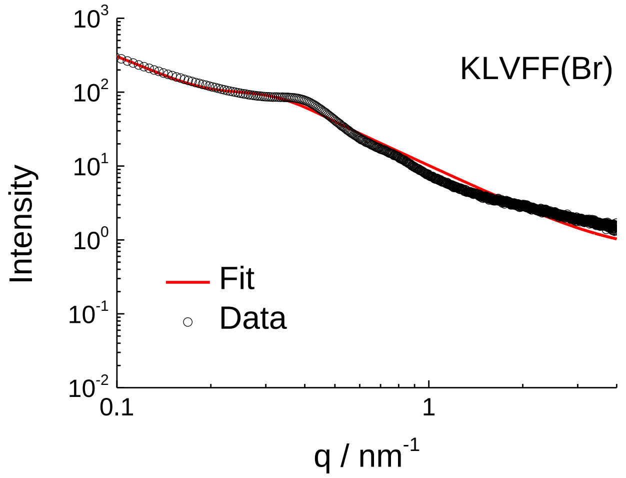

Figure S13. SAXS profile of 15 mM KLVFF(Br) dispersion with fitting analysis according to a long cylindrical shell form factor.

2.8 X-ray diffraction analysis - Structural characterization of halogenated peptides.

H_2N-Lys-Leu-Val-(*p*-IodoPhe)-(*p*-IodoPhe)-COOH i.e. KLVF(I)F(I), H_2N-Lys-Leu-Val-(*p*-BromoPhe)-(*p*-BromoPhe)-COOH, i.e. KLVF(Br)F(Br) and H_2N-Lys-Leu-Val-(*p*-ChloroPhe)-(*p*-ChloroPhe)-COOH, i.e. KLVF(Cl)F(Cl) were obtained as solvated species by dissolving the peptide in a water/hexafluoro-2-propanol 90:10 mixture. Crystals suitable for XRD analysis were obtained after two months of slow evaporation. Data collections were performed at the X-ray diffraction beamline (XRD1) of the Elettra Synchrotron, Trieste

(Italy).[4S] The crystals were dipped in perfluoropolyether vacuum oil (Fomblin) and mounted on the goniometer head with a nylon loop. Complete datasets were collected at 100 K (nitrogen stream supplied through an Oxford Cryostream 700) through the rotating crystal method. Data were acquired using a monochromatic wavelength of 0.850 Å for KLVF(I)F(I) and 0.700 Å for KLVF(Br)F(Br) and KLVF(Cl)F(Cl) on a Pilatus 2M hybrid-pixel area detector. The diffraction data were indexed and integrated using XDS.[5S] Scaling have been done using CCP4-Aimless code.[6S,7S] Crystals appear as very thin needles prone to radiation damage, as previously reported for other halogenated molecules.[8S,9S] For the brominated peptide we managed to collect a complete dataset from a unique crystal; for the iodine and chlorine derivatives, diffraction decayed even after small doses so four different datasets had to be merged for KLVF(I)F(I) and three datasets for KLVF(Cl)F(Cl) (collected from different crystals randomly oriented). Semi-empirical absorption correction and scaling was performed for the KLVF(Br)F(Br) dataset, exploiting multiple measures of symmetry-related reflections, using SADABS program.[10S] The structures were solved by the dual space algorithm implemented in the SHELXT code.[11S] Fourier analysis and refinement were performed by the full-matrix least-squares methods based on F2 implemented in SHELXL-2014.[12S] The Coot program was used for modeling.[13S]

KLVF(Cl)F(Cl) peptide crystallized in a monoclinic unit cell (P 2_1 space group). The model has been fully refined anisotropic as a 2-component non-merohedral twin. Crystal showed two domains related by a 180° rotation around the *c** reciprocal lattice direction (twin fraction refined to 17%). One peptide and four water molecules have been found in the asymmetric unit.

KLVF(Br)F(Br) and KLVF(I)F(I) peptides crystallized in equivalent conditions and showed the same P $2_12_12_1$ orthorhombic crystalline form. The cell volume is slightly bigger for the iodinated peptide, as expected from comparison of the halogen atomic radius. None of the crystals tested diffracted better than 1.1 Å, and considering radiation damage, the overall dataset resolution is not better than ~1.25 Å, for both the compounds. The number of data for model fitting was therefore limited and, to avoid over-refinement, anisotropic thermal motion modeling has been applied only to halogen atoms of the peptide (the heaviest atoms in the structures). Geometric restraints on bond lengths and angles (DFIX, DANG) have been used for all the residues and thermal motion parameters restrains (SIMU) have been applied on disordered and poorly defined fragments. Hydrogen atoms were included at calculated positions with isotropic $U_{factors}$ = 1.2 U_{eq} or $U_{factors}$ = 1.5 U_{eq} for methyl and hydroxyl

groups (U_{eq} being the equivalent isotropic thermal factor of the bonded non-hydrogen atom). R1(free) [14S] values have been calculated for the brominated and iodinated models, omitting 5% of reflections (randomly selected) from refinement cycles. Reasonable agreement of final R1(free) with R1 values (Table S1) exclude over-refinement issues, despite poor data/parameters ratios. A final refined Flack parameter 0.005(32) [15S] for KLVF(Br)F(Br) confirms the reliability of the stereochemical configuration shown. The flack parameters for KLVF(I)F(I) and KLVF(Cl)F(Cl) are not reliable as a consequence of dataset merging in presence of significant radiation damage (Flack parameter 0.521(54) and 0.38(18)). Nevertheless, R1 increases significantly inverting the structure (almost doubles) suggesting that the stereochemical configuration is the same as KLVF(Br)F(Br) (as expected from synthetic pathway). Pictures were prepared using Mercury [16S] and Pymol software.[17S] Essential crystal and refinement data (Table S4) are reported below.

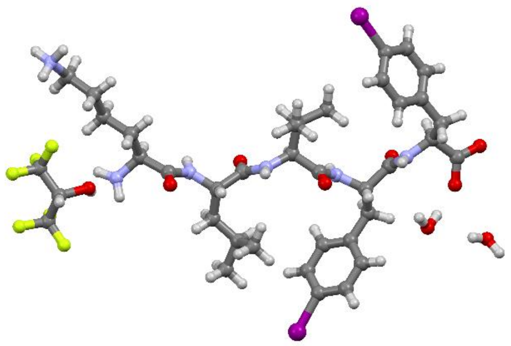

A)

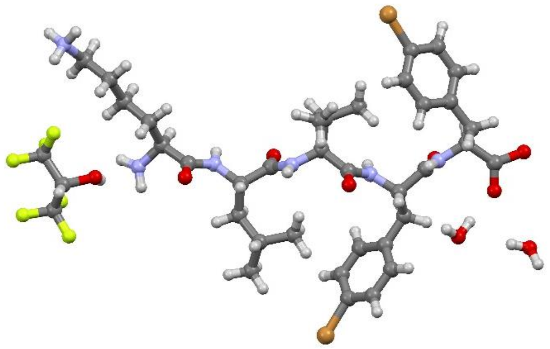

B)

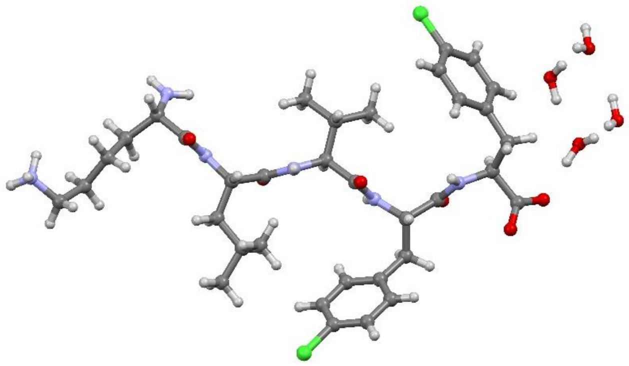

C)

Figure S14. A) Stick representation of **KLVF(I)F(I)** ASU content; B) Stick representation of **KLVF(Br)F(Br)** ASU content. C) Stick representation of **KLVF(Cl)F(Cl)** ASU content **KLVF(I)F(I)** and **KLVF(Br)F(Br)** crystallize in the same crystal form and trap one hexafluoro-2-propanol and two water solvent molecules. **KLVF(Cl)F(Cl)** crystallizes with four water molecules.

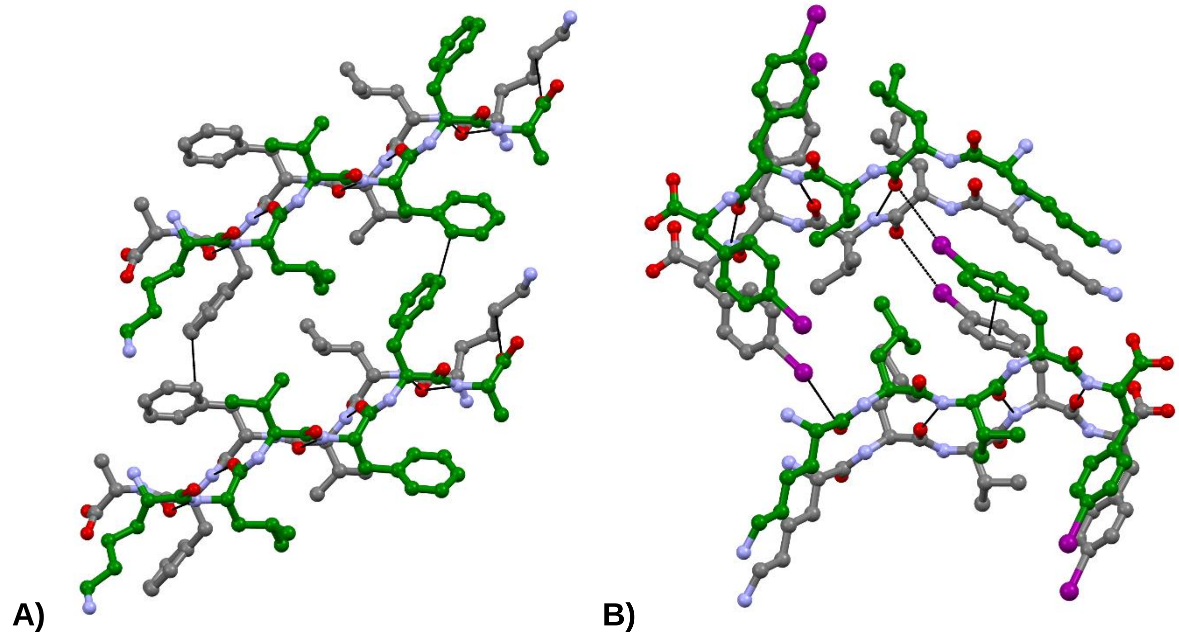

Figure S15. A) **KLVFFA** (PDB$_{ID}$ 2Y29) monomers forming an antiparallel β-sheet; B) **KLF(I)F(I)** monomers forming a parallel β-sheet.

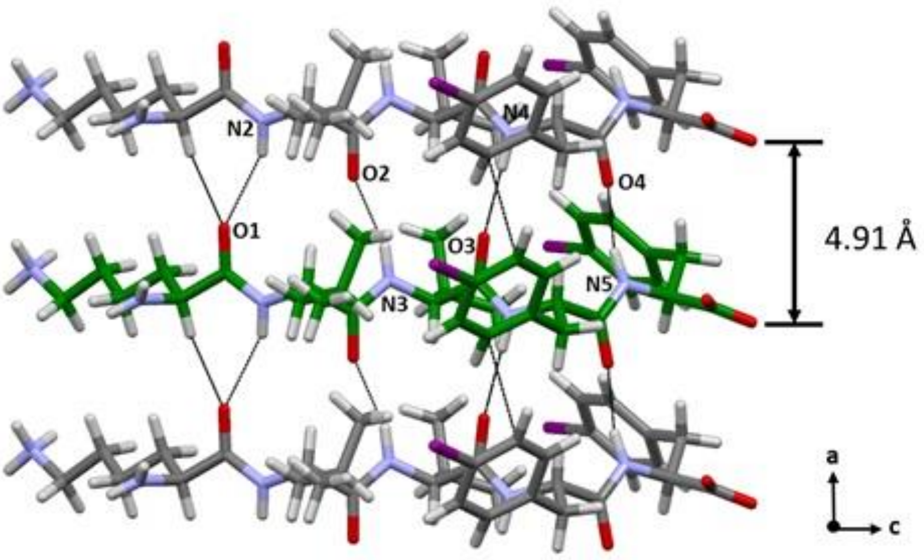

Figure S16. Crystal structure of KLVF(I)F(I). Hydrogen bonding contacts in a parallel β-sheet (Distances: N2H···O1 3.09(4) Å N3H···O2 2.99(3) Å N4H···O3 2.88(4) Å N5H···O4 2.85(4) Å) b). Color code: C, grey, green; O, red; N, violet; I, purple; F, yellow; H, white.

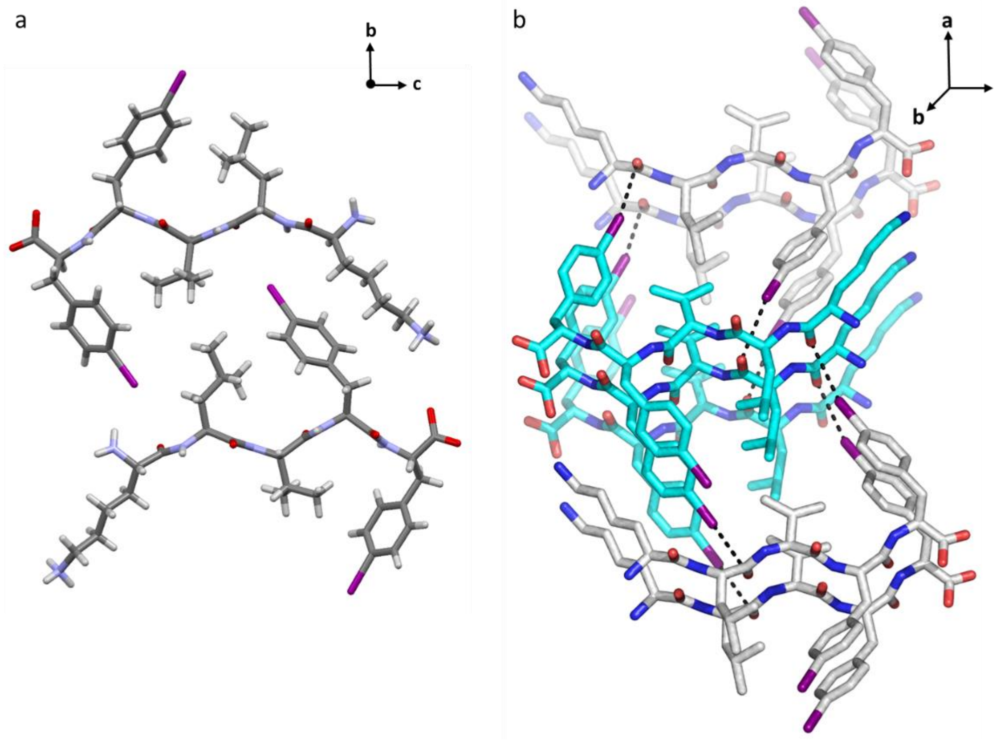

Figure S17. Overview of KLVF(I)F(I) steric zipper. View along the crystallographic a axis of two facing β-sheets showing the remarkable shape complementarity characteristic of the ‘steric zipper’ a). Staggered view of interdigitating β-sheets, with short contacts I··· O stabilizing the resulting steric zippers b). Color code: C, grey, sky blue; O, red; N, violet, blue; I, purple; H, white.

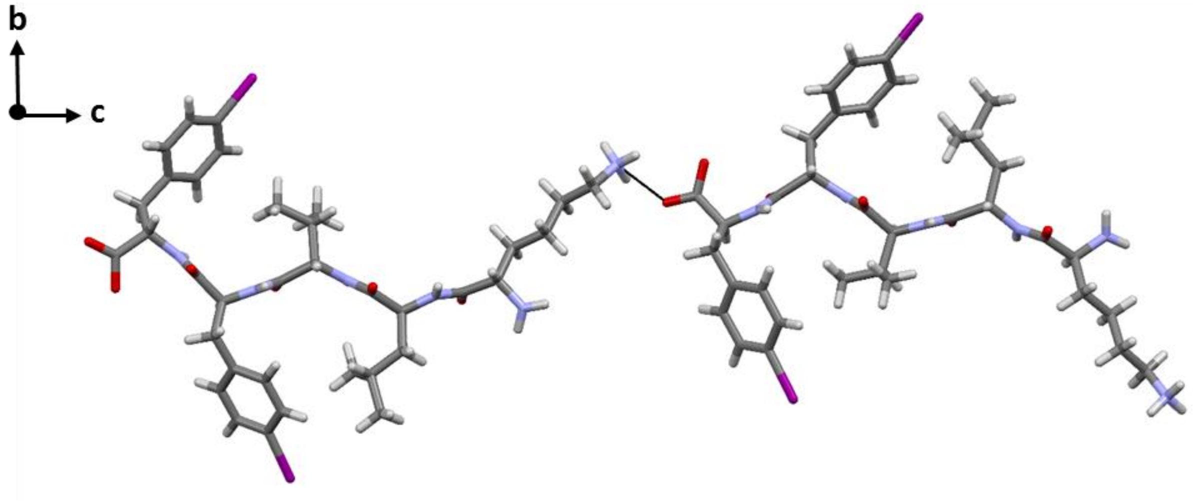

Figure S18. Lateral self-assembly of KLVF(I)F(I) driven by electrostatic interactions among the charged termini of the peptide strands (N··· O distance 2.68(6) Å). Color code: C, grey; O, red; N, violet; I, purple; H, white.

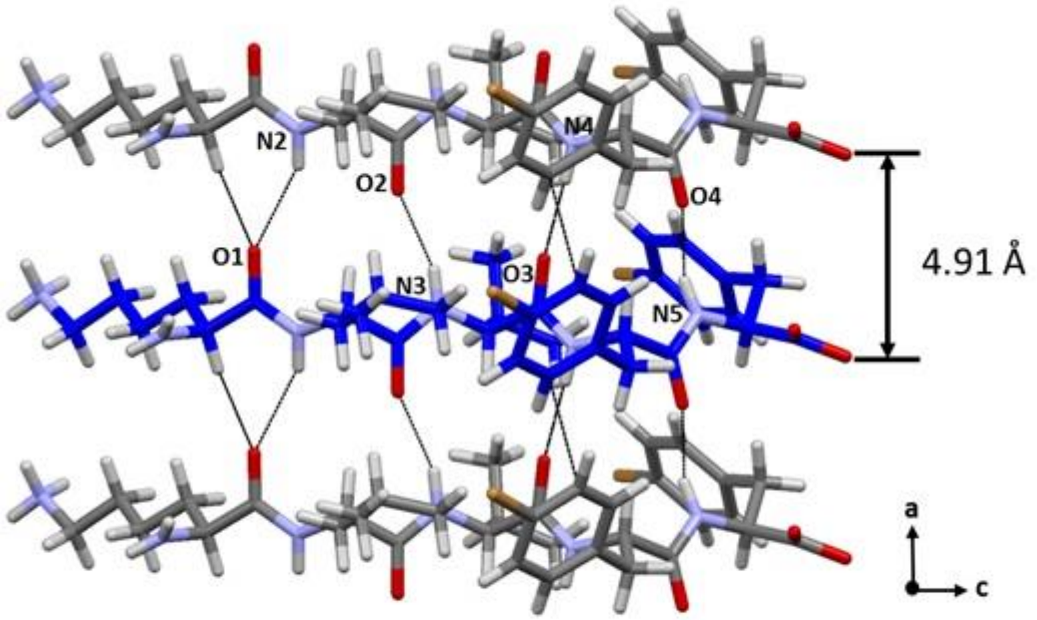

Figure S19. Crystal structure of KLVF(Br)F(Br). Hydrogen bonding contacts in a parallel β-sheet (Distances: N2H···O1 3.14(5) Å N3H···O2 3.01(4) Å N4H···O3 2.89(5) Å N5H···O4 2.84(5) Å) b). Color code: C, grey, blue; O, red; N, violet; Br, orange; F, yellow; H, white.

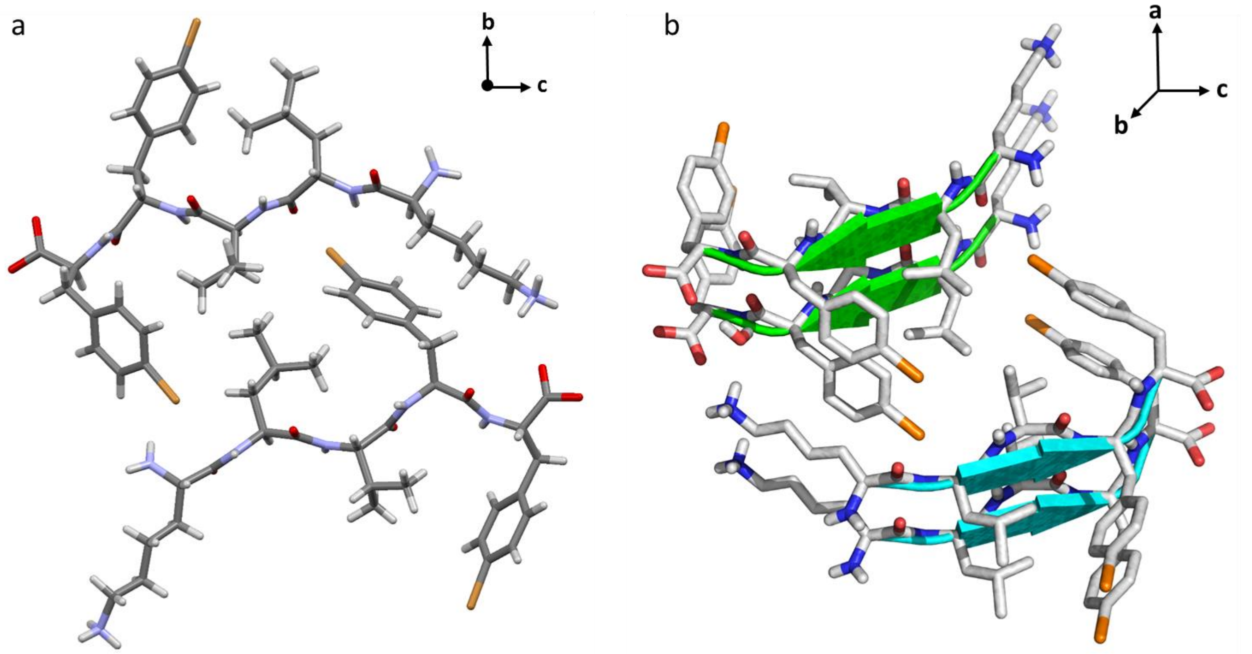

Figure S20. Overview of KLVF(Br)F(Br) steric zipper. View along the crystallographic *a* axis of two facing β-sheets showing the remarkable shape complementarity characteristic of the 'steric zipper' a). Staggered view of interdigitating β-sheets b). β-strands are depicted as cartoons. Color code: C, grey; O, red; N, violet, blue; Br, orange; H, white.

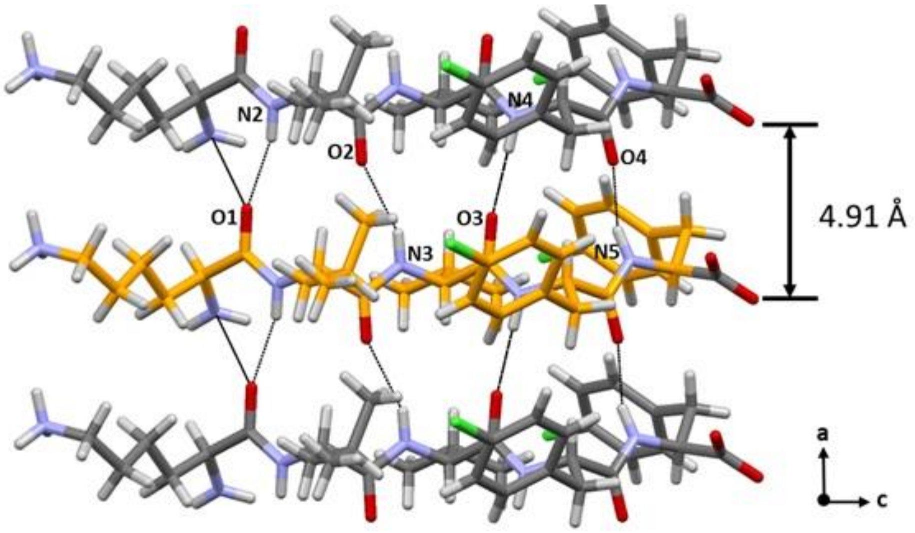

Figure S21. Crystal structure of KLVF(Cl)F(Cl). Hydrogen bonding contacts in a parallel β-sheet (Distances: N2H···O1 2.95(1) Å N3H···O2 3.09(9) Å N4H···O3 2.87(1) Å N5H···O4 2.84(1) Å) b). Color code: C, grey and orange; O, red; N, violet; Cl, green; H, white.

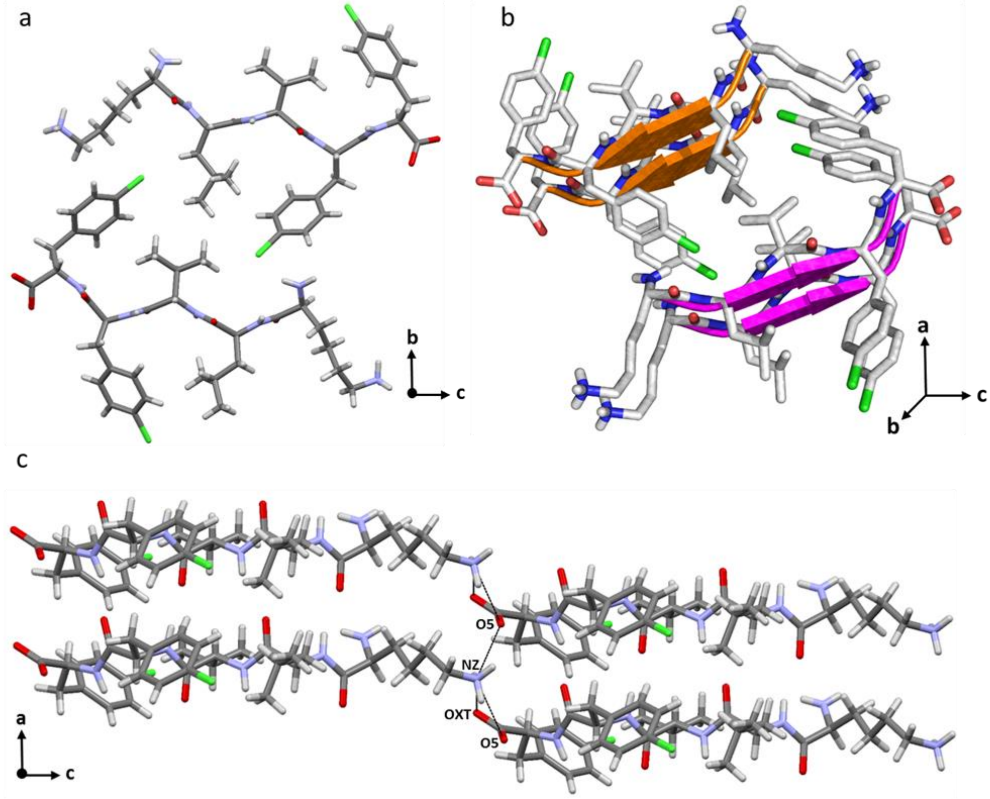

Figure S22. KLVF(Cl)F(Cl) steric zipper and lateral self-assembly. View along the crystallographic a axis of two facing β-sheets showing the remarkable shape complementarity characteristic of the 'steric zipper' a). Staggered view of interdigitating β-sheets; β-strands are depicted as cartoons b). Lateral self-assembly of KLVF(Cl)F(Cl) driven by electrostatic interactions among the charged groups of the peptide strands (NZ··· O5 distance 2.99(1) Å, 2.74(1) Å; NZ··· OXT distance 2.86(1) Å) c). Color code: C, grey; O, red; N, violet, blue; Cl, green; H, white.

2.9 Infrared spectroscopy

Infrared spectra were recorded at room temperature using a Nicolet iS50 FT-IR spectrometer equipped with a DTGS detector. Peptides were analyzed as solutions (after heating at 100 °C in order to break any pre-formed fibrils) or gels at 15 mM in D_2O. Spectra represent an average of 64 scans recorded in a single beam mode with a 4 cm^{-1} resolution and corrected for the background. The second derivative analyses of the spectra were performed using the Nicolet FTIR software, Omnic 9.0®, with a 13-point and 3rd polynomial order Savitzky and Golay function. Second derivative spectra generated negative bands as compared with the original spectra, thus for comparison all the second-derivative spectra were multiplied by -1.

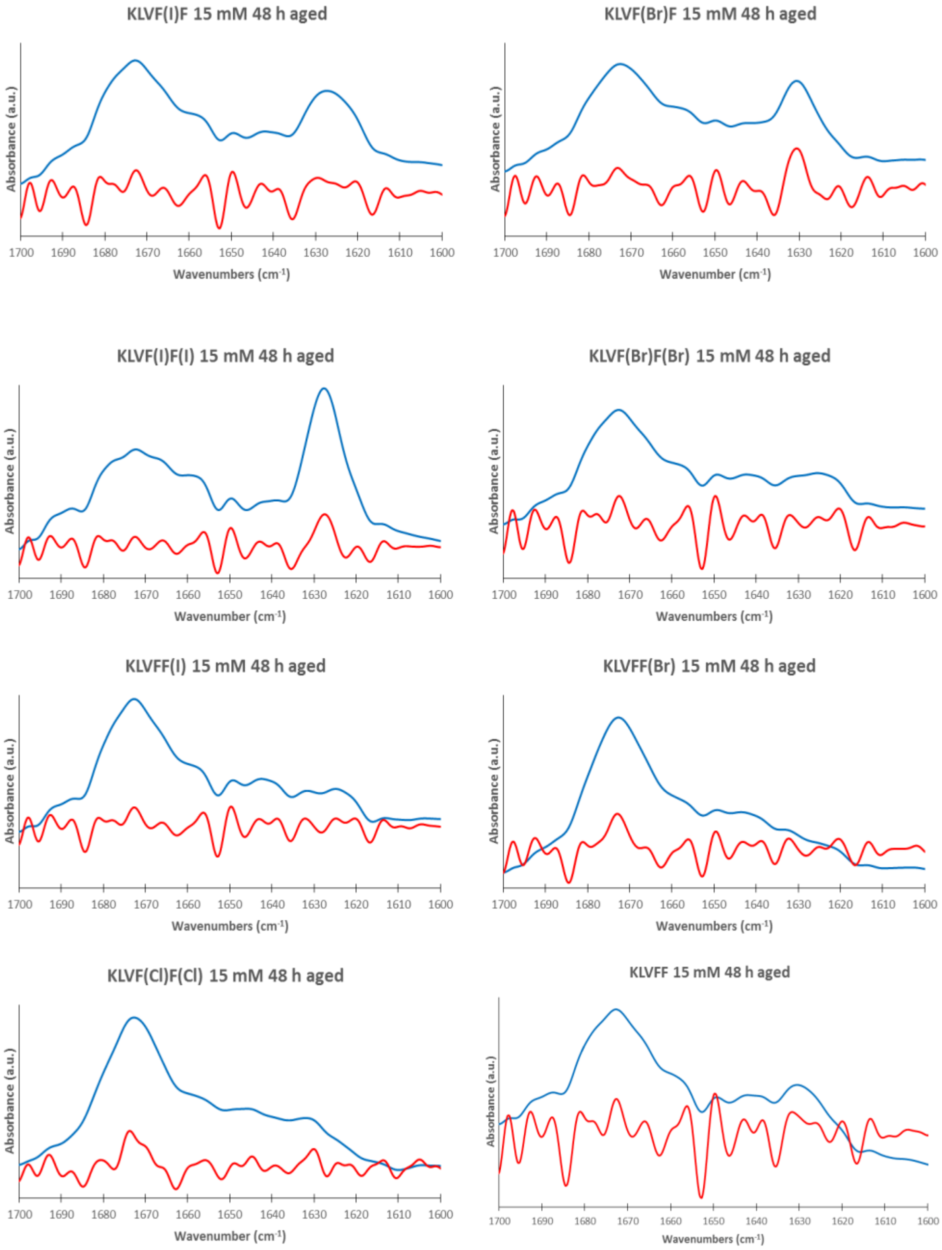

Figure S23. FTIR spectroscopy of 15 mM peptides gels/solutions after standing for 48 hours at r.t.

2.10 Circular Dichroism (CD) Spectroscopy

All the circular dichroism experiments were carried out in deionized water (18.2 MΩ·cm) in detachable quartz cuvettes, using a JASCO J-815 CD spectrometer. Acquisitions were performed between 190 and 250 nm with a 0.1 nm data pitch, 1 nm bandwidth, 100 nm min^{-1} scanning speed and 1 s response time. All the spectra are an average of 10 scans and were corrected from a reference solution, comprised of deionized water (18.2 MΩ·cm) alone. Raw data (θ, in mdeg) were subsequently converted to mean residue ellipticity ([θ] in $deg{\cdot}cm^{2}{\cdot}dmol^{-1}$) for the sake of comparison, in accordance with the following formulae:[18S]

$$[\theta] = \frac{\theta}{10 * l * c * (n-1)}$$

where θ is the observed ellipticity in mdeg, c is the concentration of the sample in $mol{\cdot}L^{-1}$, (n-1) is the number of peptide bonds, and l is the pathlength of the cuvette in cm.

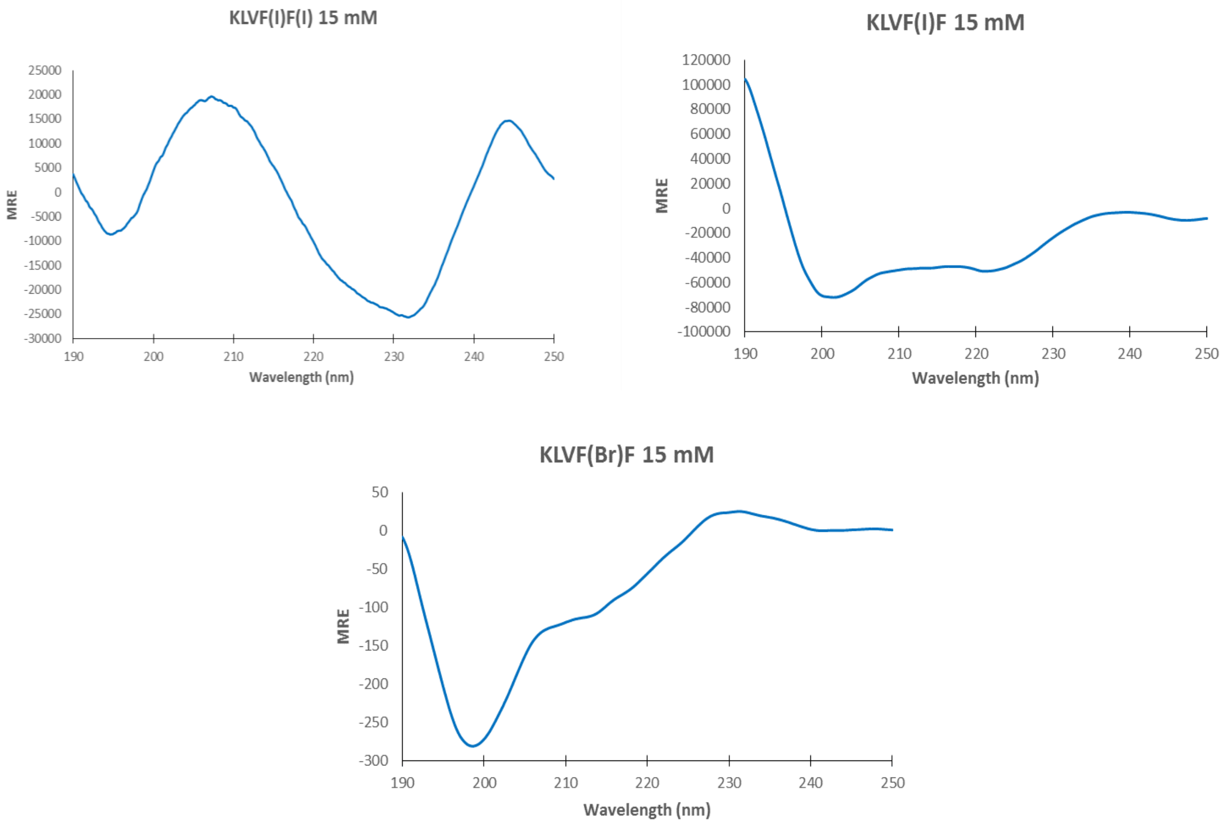

Figure S24. Circular Dichroism spectra of the forming gel-peptides. All the spectra were recorded at 15 mM concentrations. It is well known that the presence of aromatic groups in a peptide sequence can perturb CD signals related to secondary structure since n-π* and π-π* transitions between aromatic groups also absorb in the same region. It can, therefore, be not straightforward to draw definitive conclusions related to peptide secondary structure motifs from CD.

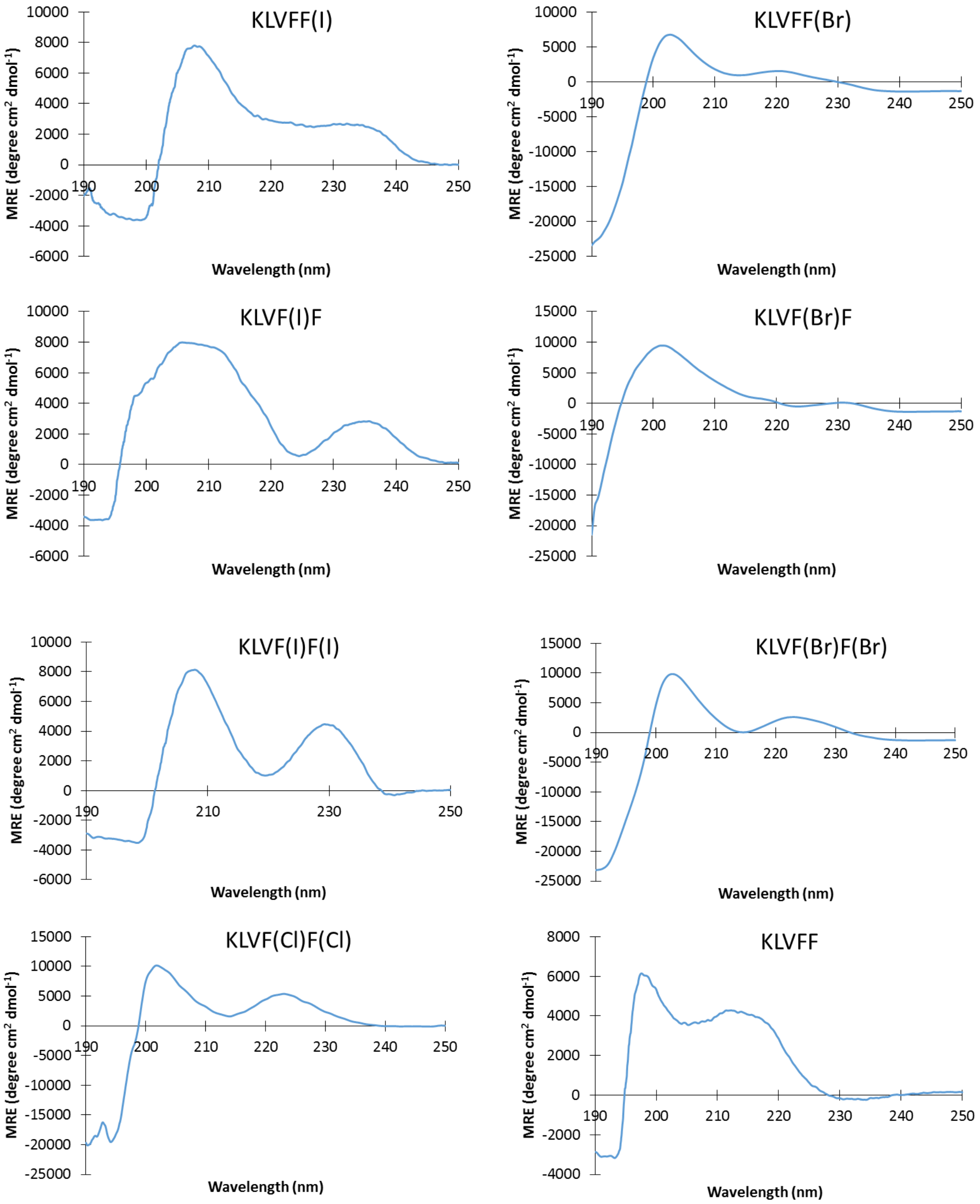

Figure S25. Circular Dichroism spectra of the KLVFF halogenated derivatives recorded in deionized water at 400 µM concentration.

2.11 Confocal Microscopy

Hydrogels were imaged using a Zeiss LSM 710 microscope with a He/Ne laser (λ_{ex}= 543 nm). The fluorescent dye, Rhodamine B, was incorporated into an aged hydrogel (48 h) scaffold by addition of 10 µl of the dye solution (0.1% w/v). Following complete absorption of the dye, the sample was excited at 543 nm and emitted light recorded using the E570LP emission filter.

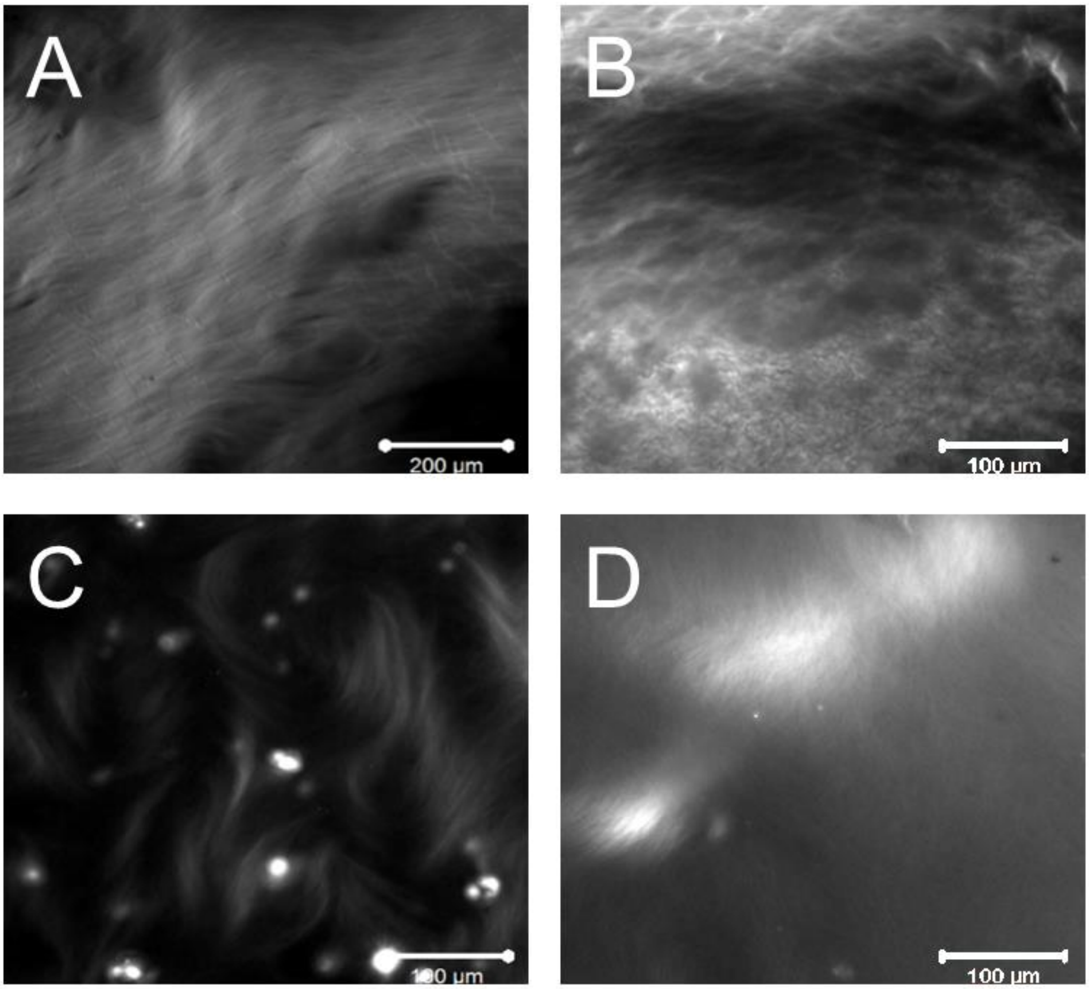

Figure S26. Confocal microscopy of KLVF(I)F after 48 h (A); KLVF(I)F after 2 weeks (B); KLVF(I)F(I) after 48 h (C); KLVF(I)F(I) after 2 weeks (D).

2.12 Congo Red Staining

All samples were monitored for green birefringence using an Olympus BX50 polarizing microscope with a SensiCam PCO camera used to display and enhance images. An 80% ethanol solution saturated with NaCl and Congo Red was freshly prepared before each measurement. A piece of each peptide hydrogel was placed on a glass microscope, allowed to air dry and then stained with Congo Red solution. Subsequently, excess Congo Red solution was blotted off the slide and the samples were analyzed using both bright and polarized light.

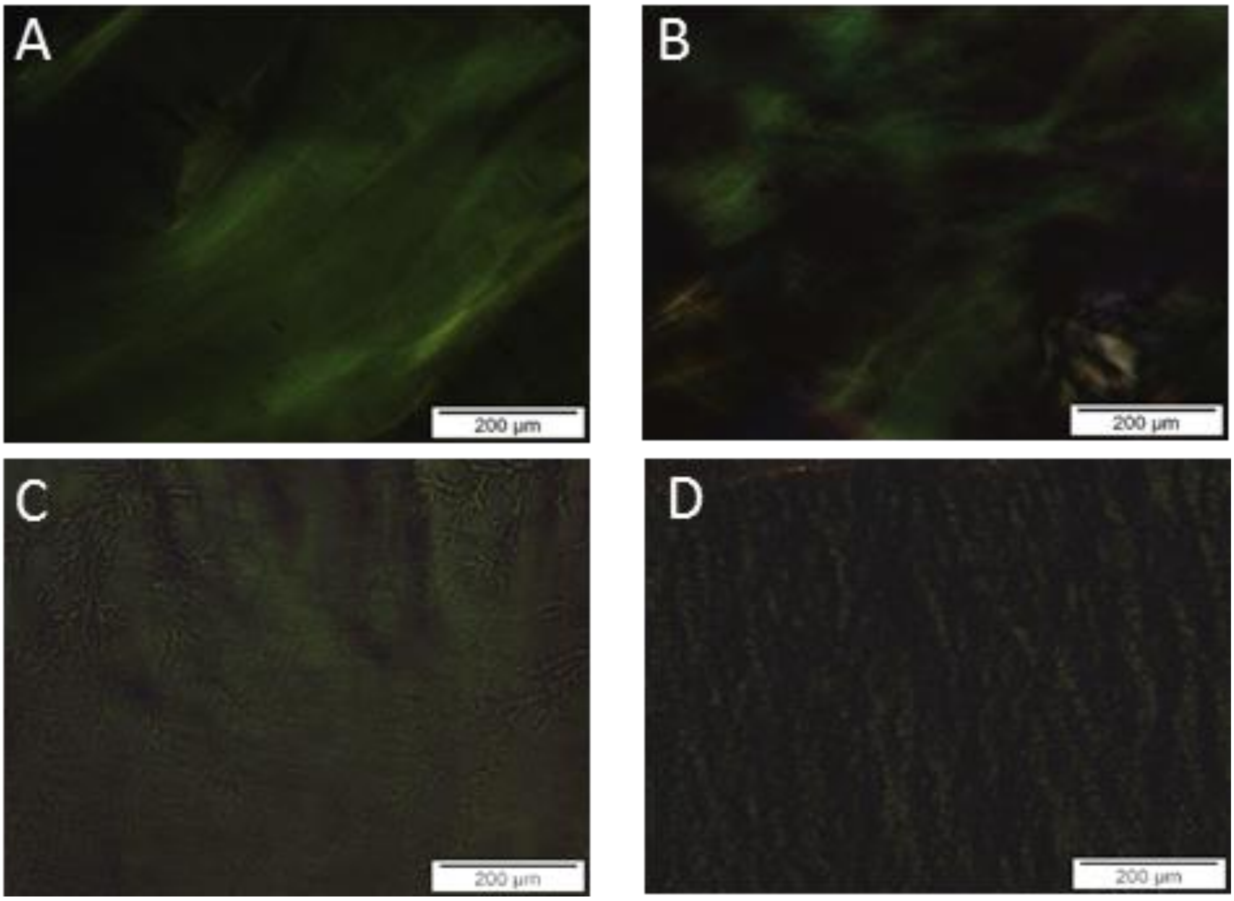

Figure S27. Congo red staining of KLVF(I)F (A); KLVF(I)F(I) (B); KLVF(Br)F (C); KLVF(Br)F(Br) (D).

2.12 Peptide synthesis

CTC Resin loading

CTC resin (400mg, 1.6 mmol/g loading) was swollen in CH_2Cl_2 for 30 min and then washed with DMF (3 × 5 mL). A solution of the entering amino acid (200□mol) and DIEA (1mmol) in DCM (4 mL) was added and the resin shaken at rt for 4 h. The resin was washed with DMF (2 × 3 mL) and capping was performed by treatment with a methanol/DIEA solution in DCM (1 x 30 min). The resin was then washed with DMF (2 × 4 mL), CH_2Cl_2 (2 × 4 mL), and DMF (2 × 4 mL). The resin was subsequently submitted to manual iterative peptide assembly (Fmoc-SPPS).

Peptide Assembly via Iterative manual SPPS

Peptides were assembled by stepwise manual Fmoc-SPPS. Activation of entering Fmoc-protected amino acids was performed using 0.5 M Oxyma in DMF / 0.5 M DIC in DMF (1:1:1 molar ratio), with a 5 equivalent excess over the initial resin loading. Capping steps were performed by treatment with a 0.3 M Ac_2O / 0.3 M DIEA solution in DMF. Fmoc- deprotection steps were performed by treatment with a 20% piperidine solution in DMF at room temperature (1 x 10 min). Following each coupling, capping or deprotection step, peptidyl-resin was washed with DMF (2 x 4 mL), DCM (1 x 4 mL) and DMF (2 x 4 mL). Upon complete chain assembly, resin was washed with DCM (5 x 4 mL) and gently dried under nitrogen flow.

Cleavage from the Resin

Resin-bound peptide was treated with an ice-cold TFA, TIS, water, thioanisole mixture (90:5:2.5:2.5 v/v/v/v, 6mL). After gently shaking the resin for 2 hours at room temperature, the resin was filtered and washed with neat TFA (2 x 4 mL). The combined cleavage solutions were worked-up as indicated below.

Work-up and Purification

Cleavage mixture was concentrated under nitrogen stream and then added dropwise to ice-cold diethyl ether (40 mL) to precipitate the crude peptide. The crude peptide was collected by centrifugation and washed with further cold diethyl ether to remove scavengers. Peptide was then dissolved in 0.1% TFA aqueous buffer (with minimal addition of ACN to aid dissolution, if necessary). Residual diethyl ether was removed by a gentle nitrogen stream and the crude peptide was purified by RP-HPLC.

RP-HPLC analysis and purification

Analytical and semi-preparative reversed phase high performance liquid chromatography (RP-HPLC) were carried out on a Tri Rotar-VI HPLC system equipped with a MD-910 multichannel detector for analytical purposes or with a Uvidec-100-VI variable UV detector for preparative purpose (all from JASCO, Tokyo, Japan). A Phenomenex Jupiter 5µ C18 90Å column (150 x 4.6 mm) was used for analytical runs and a Phenomenex Jupiter 10µ C18 90Å (250 x 21.2 mm) for peptide purification. Data were recorded and processed with Borwin software. UV detection was recorded in the 220-320 nm range. Pure RP-HPLC fractions (>97%) were combined and lyophilized.

Electro-spray ionisation mass spectrometry (ESI-MS)

Electro-spray ionization mass spectrometry (ESI-MS) was performed using a Bruker Esquire 3000+ instrument equipped with an electro-spray ionization source and a quadrupole ion trap detector (QITD).Samples were dissolved at a concentration of 0.1mg/ml in 0.1% formic acid (aq) and injected.

Synthesized peptides:

- **KLVFF**

Purification of the crude peptide by preparative RP-HPLC (10%B to 80% B over 50 min) afforded peptide KLVFF as a fluffy white solid after lyophilization. Analytical HPLC R_t 10.2 min (10 to 100 % B over 14 min, 0.1% TFA, λ= 220 nm). Mass found: $[M+1]^+$ =653.3

- **KLVFF(I)**

Purification of the crude peptide by preparative RP-HPLC (10%B to 80% B over 50 min) afforded peptide KLVFF(I) as a fluffy white solid after lyophilization. Anaytical HPLC R_t 11.7 min (10 to 100 % B over 14 min, 0.1% TFA, λ= 220 nm). Mass found: $[M+1]^+$ =779.3

- **KLVF(I)F**

Purification of the crude peptide by preparative RP-HPLC (10%B to 80% B over 50 min) afforded peptide KLVF(I)F as a fluffy white solid after lyophilization. Anaytical HPLC R_t 11.2 min (10 to 100 % B over 14 min, 0.1% TFA, λ= 220 nm). Mass found: $[M+1]^+$ =779.2

- **KLVF(I)F(I)**

Purification of the crude peptide by preparative RP-HPLC (10%B to 80% B over 50 min) afforded peptide KLVF(I)F(I) as a fluffy white solid after lyophilization. Anaytical HPLC R_t 13.0 min (10 to 100 % B over 14 min, 0.1% TFA, λ= 220 nm). Mass found: $[M+1]^+$ =904.3

- **KLVFF(Br)**

Purification of the crude peptide by preparative RP-HPLC (10%B to 80% B over 50 min) afforded peptide KLVFF(Br) as a fluffy white solid after lyophilization. Anaytical HPLC R_t 11.5 min (10 to 100 % B over 14 min, 0.1% TFA, λ= 220 nm). Mass found: $[M+1]^+$ =731.4

- **KLVF(Br)F**

Purification of the crude peptide by preparative RP-HPLC (10%B to 80% B over 50 min) afforded peptide KLVF(Br)F as a fluffy white solid after lyophilization. Anaytical HPLC R_t 11.3 min (10 to 100 % B over 14 min, 0.1% TFA, λ= 220 nm). Mass found: $[M+1]^+$ =731.2

- **KLVF(Br)F(Br)**

Purification of the crude peptide by preparative RP-HPLC (10%B to 80% B over 50 min) afforded peptide KLVF(Br)F(Br) as a fluffy white solid after lyophilization. Anaytical HPLC R_t 12.8 min (10 to 100 % B over 14 min, 0.1% TFA, λ= 220 nm). Mass found: $[M+1]^+$ =811.2

- **KLVF(Cl)F(Cl)**

Purification of the crude peptide by preparative RP-HPLC (10%B to 80% B over 50 min) afforded peptide KLVF(Cl)F(Cl) as a fluffy white solid after lyophilization. Anaytical HPLC R_t 12.6 min (10 to 100 % B over 14 min, 0.1% TFA, λ= 220 nm). Mass found: $[M+1]^+$ =721.3

2.14 Tables

Table S1. Minimum gelation concentrations of the studied peptides. The peptides showing aggregation and precipitation are not reported.

Peptide	Minimum gelation concentration (mM)	Mimimum gelation concentration (%w/w)	Time required for gelation
KLVF(I)F	7	0.5	60 h
KLVF(I)F(I)	15	1.3	15 h
KLVF(Br)F	10	0.7	72 h

Table S2. Average gelation time of the forming-gel peptides at 15 mM concentration

Peptide	Time required for gelation at 15 mM
KLVF(I)F	< 10 min
KLVF(I)F(I)	15 h
KLVF(Br)F	< 30 min

Table S3. Retention time of studied peptides in reverse-phase HPLC (data referred to purchased peptides).

Peptide	Retention time (min)
KLVFF	20.14
KLVFF(I)	22.46
KLVF(I)F	22.56
KLVF(I)F(I)	24.38
KLVFF(Br)	22.02
KLVF(Br)F	22.42
KLVF(Br)F(Br)	23.39
KLVF(Cl)F(Cl)	22.73

Table S4. Crystallographic data and refinement details for compounds **KLVF(I)F(I)** and **KLVF(Br)F(Br)**.

	**KLVF(Cl)F(Cl)**·$2H_2O$ [$C_{35}H_{50}Cl_2N_6O_6\cdot4H_2O$]	**KLVF(Br)F(Br)**·$C_3H_2F_6O\cdot2H_2O$ [$C_{35}H_{50}Br_2N_6O_6\cdot C_3H_2F_6O\cdot2H_2O$]	**KLVF(I)F(I)**·$C_3H_2F_6O\cdot2H_2O$ [$C_{35}H_{50}I_2N_6O_6\cdot C_3H_2F_6O\cdot2H_2O$]
CCDC Number	1494096	1454959	1454960

Chemical Formula	$C_{38}H_{58}Cl_2N_6O_{10}$	$C_{38}H_{56}F_6Br_2N_6O_9$	$C_{38}H_{56}F_6I_2N_6O_9$
Formula weight	793.77 g/mol	1014.70 g/mol	1108.68 g/mol
Temperature	100(2) K	100(2) K	100(2) K
Wavelength	0.700 Å	0.700 Å	0.850 Å
Crystal system	Monoclinic	Orthorhombic	Orthorhombic
Space Group	$P\ 2_1$	$P\ 2_12_12_1$	$P\ 2_12_12_1$
Unit cell dimensions	a = 4.911(1) Å<br>b = 21.326(4) Å<br>c = 19.682(4) Å<br>α = 90°<br>β = 94.49(3)°<br>γ = 90°	a = 4.909(1) Å<br>b = 20.760(4) Å<br>c = 43.408(9) Å<br>α = 90°<br>β = 90°<br>γ = 90°	a = 4.907(1) Å<br>b = 21.005(4) Å<br>c = 43.152(9) Å<br>α = 90°<br>β = 90°<br>γ = 90°
Volume	2055.0(7) $Å^3$	4423.7(15) $Å^3$	4448.2(15) $Å^3$
Z	2	4	4
Density (calculated)	1.283 $g \cdot cm^{-3}$	1.524 $g \cdot cm^{-3}$	1.656 $g \cdot cm^{-3}$
Absorption coefficient	0.207 mm^{-1}	1.846 mm^{-1}	2.388 mm^{-1}
F(000)	848	2088	2232
Crystal size	0.08 x 0.01 x 0.01 mm^3	0.08 x 0.01 x 0.01 mm^3	0.08 x 0.01 x 0.01 mm^3
Crystal habit	Colorless thin needles	Colorless thin needles	Colorless thin needles
Theta range for data collection	1.02° to 24.32°	1.34° to 16.12°	1.13° to 20.04°
Index ranges	-5 ≤ h 5,<br>-25 ≤ k ≤ 25,<br>-1 ≤ l ≤ 23	-3 ≤ h 3,<br>-15 ≤ k ≤ 16,<br>-34 ≤ l ≤ 29	-3 ≤ h 3,<br>-16 ≤ k ≤ 16,<br>0 ≤ l ≤ 34
Reflections collected	13325	4168	8932
Independent reflections	3442, 2208 data with $I>2\sigma(I)$	2225, 1400 data with $I>2\sigma(I)$	2227, 1395 data with $I>2\sigma(I)$
Data multiplicity (max resltn)	3.67 (3.55)	2.88 (3.19)	5.87 (6.19)
$I/\sigma(I)$ (max resltn)	4.56 (1.42)	5.51 (2.90)	5.62 (2.06)
R_{merge} (max resltn)	0.1537 (0.5207)	0.1173 (0.4060)	0.2072 (0.5332)
Data completeness (max resltn)	95.3% (95.1%)	97.7% (98.3%)	96.5% (94.4%)
Refinement method	Full-matrix least-squares on F^2	Full-matrix least-squares on F^2	Full-matrix least-squares on F^2
Data / restraints / parameters	3442 / 13 / 449	2225 / 185 / 255	2227 / 221 / 267
Goodness-of-fit on F^2	0.997	0.998	1.030
Δ/σ_{max}	0.012	0.005	0.009
Final R indices [$I>2\sigma(I)$]	R_1 = 0.0810<br>wR_2 = 0.1981	R_1 = 0.1397<br>wR_2 = 0.3070	R_1 = 0.0849<br>wR_2 = 0.1908
R indices (all data)	R_1 = 0.1274<br>wR_2 = 0.2337	R_1 = 0.1944, wR_2 = 0.3470<br>R_1(free) = 0.2505<br>(for 241 reflections)	R_1 = 0.1475, wR_2 = 0.2282<br>R_1(free) = 0.1577<br>(for 474 reflections)
Largest diff. peak and hole	0.561 and -0.706 $eÅ^{-3}$	0.628 and -0.638 $eÅ^{-3}$	0.860 and -0.714 $eÅ^{-3}$
R.M.S. deviation from mean	0.081 $eÅ^{-3}$	0.124 $eÅ^{-3}$	0.141 $eÅ^{-3}$

$R_1 = \Sigma \, ||Fo|-|Fc|| \, / \, \Sigma \, |Fo|$

$wR_2 = \{\Sigma \, [w(Fo^2 - Fc^2)^2] \, / \, \Sigma \, [w(Fo^2)^2]\}^{½}$

3) References

[1S] Bressler, I.; Kohlbrecher, J.; Thünemann, A. F. J. Appl. Crystallogr. 2015, 48, 1587.

[2S] Pabst, G.; Rappolt, M.; Amenitsch, H.; Laggner, P. Phys. Rev. E 2000, 62, 4000.

[3S] Castelletto, V.; Cheng, G.; Stain, C.; Connon, C. J.; Hamley, I. W. Langmuir 2012, 28, 11599.

[4S] Lausi A., Polentarutti M., Onesti S., Plaisier J. R., Busetto E., Bais G., Barba L., Cassetta A., Campi G., Lamba D., Pifferi A., Mande S. C., Sarma D. D., Sharma S. M., Paolucci G. (2015). Status of the crystallography beamlines at Elettra. The European Physical Journal Plus, 130(43), 1.

[5S] Kabsch W. (2010). XDS. Acta Crystallographica Section D, 66(2), 125.

[6S] Winn, M. D., Ballard, C. C., Cowtan, K. D., Dodson, E. J., Emsley, P., Evans, P. R., Keegan, R. M., Krissinel, E. B., Leslie, A. G. W., McCoy, A., McNicholas, S. J., Murshudov, G. N., Pannu, N. S., Potterton, E. A., Powell, H. R., Read, R. J., Vagin, A. & Wilson, K. S. (2011). Overview of the CCP4 suite and current developments. Acta Crystallographica Section D. 67, 235.

[7S] Evans P. R. and Murshudov G. N. How good are my data and what is the resolution? (2013). Acta Crystallographica Section D. 69, 1204.

[8S] Zwart, P. H., Banumathi, S., Dauter, M. & Dauter, Z. (2004). Radiation-damage-induced phasing with anomalous scattering: substructure solution and phasing. Acta Crystallographica Section D, 60, 1958.

[9S] McGeehan J. E., Carpentier P., Royant A., Bourgeois D., Ravelli R. B. G. (2007). X-ray radiation-induced damage in DNA monitored by online Raman. Journal of Synchrotron Radiation, 14, 99.

[10S] Sheldrick G. M. (2012). SADABS. University of Göttingen, Germany

[11S] Sheldrick G. M. (2015). SHELXT – Integrated space-group and crystal-structure determination. Acta Crystallographica Section A, *71*, 3.

[12S] Sheldrick G. M. (2015). Crystal structure refinement with SHELXL. Acta Crystallographica Section C, 71, 3.

[13S] Emsley P., Cowtan K. (2004). Coot: model-building tools for molecular graphics. Acta Crystallographica Section D, 60(12), 2126.

[14S] Brünger A. T. (1992). Free R value: a novel statistical quantity for assessing the accuracy of crystal structures. Nature 355, 472.

[15S] Parsons, S., Flack, H. D., Wagner, T. (2013). Use of intensity quotients and differences in absolute structure refinement. Acta Crystallographica Section B, 69(3), 249.

[16S] C. F. Macrae, I. J. Bruno, J. A. Chisholm, P. R. Edgington, P. McCabe, E. Pidcock, L. Rodriguez-Monge, R. Taylor, J. van de Streek and P. A. Wood (2008). Mercury

CSD 2.0 - New Features for the Visualization and Investigation of Crystal Structures. J. Appl. Cryst., 41, 466.

[17S] Delano, W. L. “The PyMOL Molecular Graphics System”, http://www.pymol.org 2002.

[18S] Correa, D. H. A. & Ramos, C. H. I. (2009). The use of circular dichroism spectroscopy to study protein folding, form and function. African J. Biochem. Res. 3, 164.
